# Proteomic Dissection of Nanotopography-Sensitive Mechanotransductive Signaling Hubs that Foster Neuronal Differentiation in PC12 Cells

**DOI:** 10.3389/fncel.2017.00417

**Published:** 2018-01-04

**Authors:** Elisa Maffioli, Carsten Schulte, Simona Nonnis, Francesca Grassi Scalvini, Claudio Piazzoni, Cristina Lenardi, Armando Negri, Paolo Milani, Gabriella Tedeschi

**Affiliations:** ^1^Department of Veterinary Medicine, Università degli Studi di Milano, Milan, Italy; ^2^Centre for Nanostructured Materials and Interfaces, Università degli Studi di Milano, Milan, Italy; ^3^Fondazione Filarete, Milan, Italy

**Keywords:** mechanotransduction, integrin signaling, quantitative shot gun proteomics, biophysics, biomaterial, neuronal differentiation, cell adhesion

## Abstract

Neuronal cells are competent in precisely sensing nanotopographical features of their microenvironment. The perceived microenvironmental information will be “interpreted” by mechanotransductive processes and impacts on neuronal functioning and differentiation. Attempts to influence neuronal differentiation by engineering substrates that mimic appropriate extracellular matrix (ECM) topographies are hampered by the fact that profound details of mechanosensing/-transduction complexity remain elusive. Introducing omics methods into these biomaterial approaches has the potential to provide a deeper insight into the molecular processes and signaling cascades underlying mechanosensing/-transduction but their exigence in cellular material is often opposed by technical limitations of major substrate top-down fabrication methods. Supersonic cluster beam deposition (SCBD) allows instead the bottom-up fabrication of nanostructured substrates over large areas characterized by a quantitatively controllable ECM-like nanoroughness that has been recently shown to foster neuron differentiation and maturation. Exploiting this capacity of SCBD, we challenged mechanosensing/-transduction and differentiative behavior of neuron-like PC12 cells with diverse nanotopographies and/or changes of their biomechanical status, and analyzed their phosphoproteomic profiles in these settings. Versatile proteins that can be associated to significant processes along the mechanotransductive signal sequence, i.e., cell/cell interaction, glycocalyx and ECM, membrane/f-actin linkage and integrin activation, cell/substrate interaction, integrin adhesion complex, actomyosin organization/cellular mechanics, nuclear organization, and transcriptional regulation, were affected. The phosphoproteomic data suggested furthermore an involvement of ILK, mTOR, Wnt, and calcium signaling in these nanotopography- and/or cell mechanics-related processes. Altogether, potential nanotopography-sensitive mechanotransductive signaling hubs participating in neuronal differentiation were dissected.

## Introduction

Cells can sense nanotopographical cues deriving from the extracellular matrix (ECM), predominantly through integrin adhesion complexes (IAC), and the microenvironmental information is converted into alterations of the cytoskeletal organization and mechanics. Mechanosensitive signaling cascades and nuclear rearrangements eventually translate these modulations into cellular responses; this entire sequence is defined as mechanotransduction. Mechanosensing of the substrate nanotopography and the subsequent mechanotransduction strongly impact many physiological cellular behaviors, in particular in the context of cell differentiation (Geiger et al., 2009; Wang et al., 2009; Chen et al., 2014; Dalby et al., 2014; Jansen et al., 2015), but they might also influence pathophysiological processes, such as metastatic cell migration (Park et al., 2016).

Many aspects regarding the cellular capacity of sensing nanoscale topographical cues and how the information is integrated into complex mechanotransductive signals are still largely unknown (Chen et al., 2014; Dalby et al., 2014). Artificial nanostructured surfaces produced by diverse top-down microfabrication techniques (typical of the semiconductor industry) have been useful tools that helped to understand principal surface topography-related parameters controlling mechanotransductive processes (Chen et al., 2014; Dalby et al., 2014) which are hard to access *in vivo*. This approach has two major limitations. First of all, starting from simple surface motifs it is extremely difficult to reconstruct the morphological complexity of the ECM. Secondly, achieving an in-depth comprehension of the mechanotransductive processes and signaling requires systematic high-throughput and omic approaches (Cranford et al., 2013; Groen et al., 2016). Admittedly, many micro-/nanofabrication techniques have technical limitations hindering a feasible and cost-effective scale-up necessary to provide sufficiently large surface areas with a defined nanotopography (Chen et al., 2014). However, the yield of a reasonable amount of cellular material is mandatory for the implementation of omics approaches.

In this framework, we use the bottom-up nanofabrication method supersonic cluster beam deposition (SCBD) to quantitatively address the influence of nanoscale surface topography on mechanotransduction. SCBD permits the engineering of nanostructured surfaces with a reproducible nanoscale roughness parameter by assembling transition metal oxide clusters (Schulte et al., 2017), thereby realizing topographies that mimic ECM nano-features (Gasiorowski et al., 2013). SCBD can be applied efficiently to produce biocompatible substrates (made by titania or zirconia clusters) on large macroscopic areas rendering it compatible with profound biological analyses, such as proteomic studies (Schulte et al., 2017).

Recently, using PC12 cells as a broadly accepted model system for neuronal differentiation, we demonstrated that appropriate biophysical stimuli; provided by the cellular interaction with nanotopographical cues of titania or zirconia surfaces produced by SCBD, promote neuronal differentiation processes (Tamplenizza et al., 2013; Schulte et al., 2016a). This potential of the cluster-assembled surfaces was also confirmed in hippocampal neurons (Schulte et al., 2016b). In both cellular models we applied label-free shotgun proteomics as an essential technique to examine the impact of the nanotopography on cellular differentiation processes since this quantitative approach can achieve simultaneously: (a) the identification of thousands of proteins isolated from a cellular model and (b) the quantification of each protein/phosphosite. It is therefore well suited to study differences in global protein expression between different samples, providing substantial information to delineate and profoundly understand cell signaling pathways and modulations of the cellular program (Toffolo et al., 2014; Zanotti et al., 2016), in particular also in the context of integrin-mediated mechanotransduction (Humphries et al., 2015; Robertson et al., 2015).

Neuronal differentiation is a specifically interesting biological process in this mechanobiological context. It is accompanied by drastic morphological and cytoskeletal rearrangements throughout the realization of neurites, dendrites and axons, strongly controlled by point contact-mediated neuron/ECM interaction (Myers et al., 2011; Flynn, 2013; Kerstein et al., 2015). In fact, in PC12 cells the extent of nanotopography-triggered differentiation (even in the absence of a biochemical stimulus) was comparable to the canonical differentiation mediated by NGF-induced TrkA activation. In each case, either NGF- or nanotopography-induced, the outcome was the outgrowth of neurites and a differentiated cell. Our previous study furthermore revealed that in the latter condition, complex mechanotransductive events were at the basis of cellular processes that lead to the onset of neuritogenesis and neuronal differentiation. However, at the proteome level we only compared the nanostructured surface with a roughness parameter R_*q*_ of 15 nm root mean square (RMS) (which induced the strongest neuritogenesis, called ns-Zr15 hereafter) against a flat zirconia surface (which even after NGF stimulus impeded neuronal differentiation, named flat-Zr herafter). The cells on ns-Zr15 were found to have small IAC (predominantly focal contact (FC) size), few to none stress fibers and a low cell rigidity, contrary to the large IAC (focal adhesion (FA) size), stress fibers and an increased cellular rigidity on flat-Zr (Schulte et al., 2016a).

**Figure 1:**
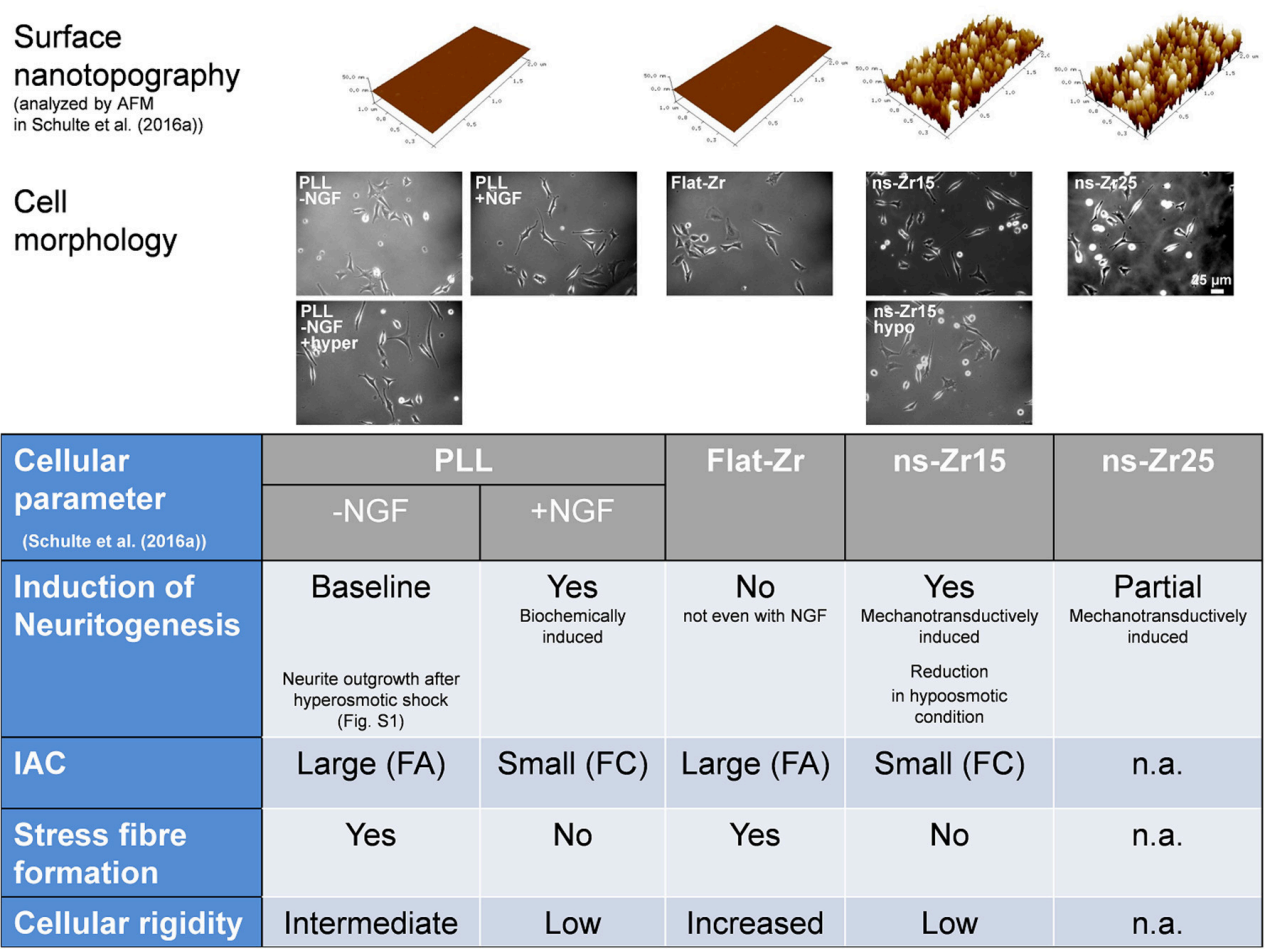
Representations of the cell morphology in the different conditions and a summary of the results presented in a previous publication (Schulte et al., 2016a). The figure summarizes the results of our previous publication (Schulte et al., 2016a) which provided the basis for the selection of the experimental conditions of the extended phosphoproteomic analyses of this work. In the upper row representations of the surface nanotopographies are displayed which were also used in this work (PLL-coated glass, flat-Zr, ns-Zr15, and ns-Zr25). Underneath example photos demonstrate the morphology of PC12 cells in the indicated conditions. In the table the impact of these different conditions on examined cellular parameter are recapitulated (FA, focal adhesions; FC, focal complexes; IAC, integrin adhesion complex; n.a., not analyzed).

Besides these two conditions (ns-Zr15 and flat-Zr), the proteomic analyses in this study comprise instead PC12 in more versatile experimental conditions including a surface nanotopography with higher roughness, the biochemically NGF-induced canonical neuronal differentiation and manipulations that affect the biomechanical status of the PC12 cell. The characteristics of these additional experimental conditions evaluated in this extended proteomic analyses are (summarized in Figure 1):

(1) The surface with an increased roughness R_*q*_ of 25 nm RMS (ns-Zr25) has asperities that display subtle differences in diameter and dimension compared to ns-Zr15. This roughness induced neuritogenesis to a lower extent with respect to ns-Zr15 (Schulte et al., 2016a).
(2 and 3)As canonical reference, representing the broadly studied biochemically triggered neuritogenesis and neuronal differentiation, PC12 cells on PLL-coated glass, in the absence (PLL) or presence of NGF (NGF), were introduced into the analysis. The cells exhibited large IAC (FA size), stress fibers and an intermediate cell rigidity without the NGF stimulus. The IAC size (to FC), stress fibers frequency and cellular rigidity decreased upon NGF-induced differentiation (Schulte et al., 2016a).
  Moreover, the role of cellular biomechanics in this mechanotransductive sequence was approached by adding two conditions affecting the cellular tension:
(4) Cells grown on ns-Zr15 in hypoosmotic medium to increase the cellular tension by osmotic swelling which counteracts the lower rigidity of the cells on ns-Zr15 and inhibits the nanotopography-triggered neuritogenesis (Schulte et al., 2016a).
(5) Cells on PLL exposed to a short hyperosmotic shock (decrease in cellular tension), a treatment that morphologically triggered the outgrowth of neurites (Figure S1).

A correlation of these alterations in cellular and topographical characteristics to changes in the PC12 neuronal proteome allowed us to obtain a deeper understanding of cellular nanotopography sensing and mechanotransductive signal integration. We were able to define potentially relevant surface nanotopography-sensitive mechanotransductive proteins and signaling networks/hubs that might play a key role in the signal integration driving, in this case, neuronal differentiation. For the sake of clarity (considering the many diverse conditions), in the main text we will talk more generally about signaling pathways and cellular processes affected or modulated in the different experimental settings, highlighting only a few particularly interesting proteins dissected from the global picture that introduce new aspects. Further examples of proteins and information on their to-date reported functions relevant in this context will be provided in the corresponding tables we refer to. The precise analysis of biomolecular events triggered by cell/nanotopography interaction, combined with the technical capacities of SCBD, constitutes the necessary foundation for efficient near-future exploitation of SCBD for versatile bio-applications.

## Experimental procedures

### Substrate preparations

As a basis for all substrates standard microscope glass slides with the dimensions of 76 × 26 mm (surface area ~20 cm^2^) were used.

On this carrier, we produced the cluster-assembled nanostructured films by supersonic cluster beam deposition (SCBD) of zirconia clusters obtained through a pulsed microplasma cluster source. Specific details on this bottom-up nanofabrication approach can be found in Wegner et al. (2006) and Schulte et al. (2017). These cluster-assembled zirconia surfaces are given the abbreviation ns-Zr throughout the manuscript. The number after Zr indicates the roughness parameter R_q_. Two batches were fabricated for this work with roughness parameters of 15 nm RMS (ns-Zr15) and 25 nm RMS (ns-Zr25). The roughness and the morphological parameters have been systematically characterized by atomic force microscopy (AFM) (Podestà et al., 2015; Borghi et al., 2016). The capacity of SCBD to reliably cover large macroscopic areas with nanostructured films of a predefined roughness allowed us to perform the experiments on microscope glass slides with the dimensions of 76 × 26 mm (~20 cm^2^ surface area). This rendered possible the yield of sufficient cellular material from the different experimental situations to obtain the analyzed information, e.g., also data on the phosphorylation status of the proteins.

The flat zirconia surfaces (flat-Zr) with a roughness of ~0.4 nm RMS were obtained with electron beam evaporation. For the canonical reference, the microscope glass slides were coated with poly-L-lysine (PLL) (Sigma-Aldrich, St. Louis, USA, Missouri) for 30 min at room temperature (RT), after cleaning with 70% ethanol and washing twice with PBS. This coating was done directly before plating the cells.

All substrates were sterilized with UV light for 10 min before seeding the cells.

### Cell culture and preparation of the cells for the experiments

PC12 cells (PC-12 Adh ATCC Catalog no. CRL-1721.1™) were routinely kept in culture in RPMI-1640 medium (Sigma-Aldrich) which was supplemented with 10% horse serum (HS, Sigma-Aldrich), 5% fetal bovine serum (FBS, Sigma-Aldrich), 2 mM L-glutamine, 10 mM HEPES, 100 units/ml penicillin, 1 mM pyruvic acid, and 100 μg/ml streptomycin. The culture conditions were 37°C and 5% CO_2_ (98% air-humified). Subculturing was performed every 2–3 days by detaching the cells with 1 mM EDTA in HBSS or a trypsin solution (Sigma-Aldrich), centrifugation at 1,000× g (5 min) and resuspension in the culture medium.

For the experiments the PC12 cells were detached with 1 mM EDTA in HBSS and centrifuged at 1,000× g (5 min), washed with low serum medium (RPMI-1640 with all the supplements, but only 1% HS and without FBS), and centrifuged again at 1,000× g (5 min). Before plating the cells on the different substrate conditions, the cells were counted with an improved Neubauer chamber and then seeded with the concentration of ~4,000 cells/cm^2^ (after resuspension in RPMI low serum medium) onto the microscope slides that were placed into non-treated 4 well dishes with the dimensions 127.8 × 85.5 mm (Thermo Fisher).

For the NGF condition, the NGF stimulus (human NGF-β, Sigma-Aldrich) was added to the medium right after plating the cells making a final concentration of 50 ng/ml. For the ns-Zr15 hypo condition, the cells were re-suspended in RPMI low serum medium diluted 7.5/2.5 with deionised water (supplements were kept at the aforementioned concentrations) and pre-incubated in the hypoosmotic medium for 15 min before plating the cells eventually into the well, always in the hypoosmotic medium. For the PLL hyper condition, after the adhesion of the cells (1 h after plating) a hyperosmotic shock was applied to the cells (150 mM sucrose final concentration in the RPMI low serum medium) for 15 min, and washed once with RPMI low serum medium. The cells were left in RPMI low serum medium for the rest of the experiment.

The cells were left in the incubator for 24 h in all conditions. After washing twice with PBS, the cellular material was yielded for the proteomic analysis by scratching the cells from the microscope slides with cell scrapers (TPP, Trasadingen, Switzerland) in the presence of icecold PBS supplemented with protease (Roche, Basel, Switzerland) and phosphatase inhibitors (phosphatase inhibitor cocktail (Cell Signaling Technology), calyculine A (serine/threonine phosphatase inhibitor) 10 nM (Cell Signaling Technology), microcystin-LR 10 nM (Enzo Life Sciences).

For the inhibition experiments with SKF96365 (Sigma-Aldrich) and GsMTx4 (Alomone Labs, Israel), the resuspended cells were preincubated with the inhibitors (SKF96365 15 μM; GsMTx4 10 μM) in RPMI low serum medium (supplemented with 50 ng/ml NGF in the PLL +NGF condition) for 15 min in suspension before plating. The inhibitor treatment was maintained for 1 h, and then the medium was discarded and exchanged with new RPMI low serum medium (plus 50 ng/ml NGF in the PLL +NGF condition). For the rapamycin inhibition (Sigma-Aldrich), the cells were treated with the indicated rapamycin concentrations for the whole duration of the experiment. After 24 h, respectively 48 h for the rapamycin experiments, the morphology of the PC12 cells was recorded with an inverted Axiovert 40 CFL microscope (Zeiss, Oberkochen, Germany) equipped with LD A-Plan 20x/0.3 Ph1 or CP-ACHROMAT 10x/0.25 Ph1 objectives (both Zeiss) and the analysis was performed with ImageJ (NIH, New York, USA). Cells with neurites >10 μm were counted as differentiated and only neurites with a length >10 μm were considered for neurite length quantification. If cells have multiple neurites only the longest two were taken into the quantification, and in case of neurite branching the longest branch was measured. The neurite morphology was comparable between the canonical biochemically (NGF-)induced and the nanotopography-triggered neuritogenesis with 1.82 ± 0.42 neurites per cell for the first and 1.66 ± 0.21 for the latter (in total 160 differentiated cells for each condition were quantified from 8 independent experiments) (Figure S2). In both cases the median was 2 neurites per cell and the vast majority of cells bore 1 or 2 neurites (together 82%, respectively 90%).

All the inhibition experiments were performed on coverslips with a diameter of 13 mm. The substrate preparation itself was the same as in the precedent section.

### Shotgun mass spectrometry and label free quantification

After reduction and derivatisation, the proteins were digested with trypsin sequence grade (Roche) for 16 h at 37°C using a trypsin:protein ratio of 1:20. LC-ESI-MS/MS analysis was performed on a Dionex UltiMate 3000 HPLC System with a PicoFrit ProteoPrep C18 column (200 mm, internal diameter of 75 μm) (New Objective, USA). Gradient: 1% ACN in 0.1% formic acid for 10 min, 1–4% ACN in 0.1% formic acid for 6 min, 4–30% ACN in 0.1% formic acid for 147 min and 30–50% ACN in 0.1% formic for 3 min at a flow rate of 0.3 μl/min. The eluate was electrosprayed into an LTQ Orbitrap Velos (Thermo Fisher Scientific, Bremen, Germany) through a Proxeon nanoelectrospray ion source (Thermo Fisher Scientific). The LTQ-Orbitrap was operated in positive mode in data-dependent acquisition mode to automatically alternate between a full scan (350–2,000 m/z) in the Orbitrap (at resolution 60000, AGC target 1000000) and subsequent CID MS/MS in the linear ion trap of the 20 most intense peaks from full scan (normalized collision energy of 35%, 10 ms activation). Isolation window: 3 Da, unassigned charge states: rejected, charge state 1: rejected, charge states 2+, 3+, 4+: not rejected; dynamic exclusion enabled (60 s, exclusion list size: 200). Four technical replicate analyses of each sample were performed. Data acquisition was controlled by Xcalibur 2.0 and Tune 2.4 software (Thermo Fisher Scientific). Mass spectra were analyzed using MaxQuant software (version 1.3.0.5). The initial maximum allowed mass deviation was set to 6 ppm for monoisotopic precursor ions and 0.5 Da for MS/MS peaks. Enzyme specificity was set to trypsin, defined as C-terminal to arginine and lysine excluding proline, and a maximum of two missed cleavages were allowed. Carbamidomethylcysteine was set as a fixed modification, while N-terminal acetylation, methionine oxidation and Ser/Thr/Tyr phosphorylation were set as variable modifications. The spectra were searched by the Andromeda search engine against the rat Uniprot sequence database (release 29.05.2013). Protein identification required at least one unique or razor peptide per protein group. Quantification in MaxQuant was performed using the built in XIC-based label free quantification (LFQ) algorithm using fast LFQ. The required false positive rate (FDR) was set to 1% at the peptide, 1% at the protein and 1% at the site-modification level, and the minimum required peptide length was set to 6 amino acids.

The mass spectrometry proteomics data have been deposited to the ProteomeXchange Consortium via the PRIDE (Vizcaíno et al., 2016) partner repository with the dataset identifier PXD007644.

### Statistical and bioinformatics analyses

Statistical analyses were performed using the Perseus software (version 1.4.0.6, www.biochem.mpg.de/mann/tools/). Only proteins/phosphopeptides present and quantified in at least 3 out of 4 technical repeats were considered as positively identified in a sample and used for statistical analyses. An Anova test (Permutation based FDR 0.05) was carried out to identify proteins/phosphopeptides differentially expressed among the different conditions. To tackle specific biological issues we then compared subsets of three proteomic data related to specific conditions, namely: [ns-Zr15, NGF, PLL], [ns-Zr15, NGF, flat-Zr], [ns-Zr15, NGF, ns-Zr25]. Therefore, proteins/phosphopeptides were considered differentially expressed if they were present only in one condition or showed a *Post-hoc* Bonferroni test *p* < 0.0167.

Regarding the proteomic data of ns-Zr15 hypo and PLL hyper which refer to peculiar cell conditions, the following comparisons were performed: [ns-Zr15, ns-Zr15 hypo], [PLL hyper and PLL], and [ns-Zr15 hypo, PLL hyper]. Proteins/phosphopeptides were considered differentially expressed if they were present only in one condition or showed a significant Welch *t*-test difference (cut-off at 5% permutation based FDR).

Bioinformatic analyses were carried out by DAVID software (release 6.7) (Huang et al., 2009), Panther software (Mi et al., 2017), ClueGO application of Cytoskape software (release 3.2.0) (http://www.cytoscape.org/), and Ingenuity Pathway Analysis (IPA®) (QIAGEN Redwood City, www.qiagen.com/ingenuity) to cluster enriched annotation groups of Molecular Function, Biological Processes, Pathways, and Networks within the set of identified proteins/phosphopeptides. The compared data sets are indicated in the relative figures. Functional grouping was based on a Fisher Exact test *p* ≤ 0.05 and at least two counts.

## Results and discussion

### Similarities and differences between biochemically and mechanotransductively promoted neuronal differentiation at the protein level

#### Focus on ns-Zr15, NGF, PLL, flat-Zr

The versatile conditions included in the extended proteomic approach presented here are summarized in the introduction and in Figure 1. Altogether, they address different aspects of surface nanotopography of the substrate and/or biomechanics of the cell, integrating also information on the phosphorylation status of the proteins.

To dissect similarities and differences between the biochemically and mechanotransductively promoted neuronal differentiation at the proteome level we compared the data sets of ns-Zr15 (neuritogenesis-triggering cluster-assembled zirconia surface), PLL and NGF [canonical condition on PLL-coated glass, in the presence (NGF) or absence (PLL) of NGF]. The Venn diagram (Figure S3A), the work flow (Figure S3B), the Volcano plots (Figure S3C) and the corresponding lists of differently expressed proteins (Tables S1–S6) are reported in the indicated Supplementary Information.

The proteomic analysis of NGF_vs_PLL (Table S1) compared to ns-Zr15_vs_PLL (Table S3) highlights the common outcome of neuronal differentiation, independent of whether initiated canonically by NGF stimulation (NGF) or instead by mechanotransductive processes (ns-Zr15). 11 out of 35 proteins found to be significantly altered in NGF_vs_PLL are differentially expressed in the same manner also in ns-Zr15_vs_PLL (the common proteins are marked in gray in Table S3). Several of these proteins indeed have prominent and versatile known roles in the regulation of neuronal functioning and neurogenic processes [such as e.g., Htra1 (Launay et al., 2008; Tennstaedt et al., 2012); Vps35 (Wang et al., 2012; Tang et al., 2015); Fasn (Knobloch et al., 2013); Pdia3/ERp57 (Castillo et al., 2015; Bargsted et al., 2016); C3 (Stevens et al., 2007); RPL19 (Zhou et al., 2010); details in Table 1: Similarities].

**Table 1:** Similarities between biochemically and mechanotransductively promoted neuronal differentiation at the protein level by comparing the conditions ns-Zr15, NGF, and PLL.

**Similarities**	**Associated to** **Similarities and differences between biochemically and mechanotransductively promoted neuronal differentiation at the protein level**			
Examples of proteins differentially expressed in the same manner in NGF_vs_PLL (Table S1) and ns-Zr15_vs_PLL (Table S3) and their reported roles in a neuronal context				
**Protein name**	**NGF and ns-Zr15**	**Reported protein functions**	**References**	**Category**
Htra1 (high temperature responsive antigen 1)	up	Htra1 is a microtubule-associated serine protease that has been found to be crucial for neuronal protein quality control (e.g., tau), survival and maturation.	Launay et al., 2008; Tennstaedt et al., 2012	Neuronal protein quality control
Vps35 (vacuolar protein sorting-associated protein 35)	up	Vps35 belongs to the retromer complex and contributes therefore essentially to endosomal trafficking. It promotes e.g., neuronal morphogenesis in hippocampal neurons by contributing to the retrograde trafficking of BACE1. In addition, mitochondria were found fused and dysfunctional in Vps35-deficient dopaminergic neurons, causing the loss of these neurons.	Wang et al., 2012; Tang et al., 2015	Vesicle trafficking
Fasn (fatty acid synthase)	up	Fasn is essential in the lipogenesis control of neural stem cells and its deletion impairs adult neurogenesis.	Knobloch et al., 2013	Lipogenesis
Pdia3 (protein disulfide-isomerase 3)/ERp57	up	This disulfide isomerase belongs to the endoplasmic reticulum proteostasis network with a neuroprotective function against misfolded prions. It is involved in axonal regeneration after peripheral nerve injury.	Castillo et al., 2015; Bargsted et al., 2016	Proteostasis network
C3 (complement component 3)	down	C3 is downregulated in the adult central nervous system and known to participate in synapse elimination.	Stevens et al., 2007	Complement system
RPL19 (60S ribosomal protein L19)	up	RPL19 was recommended as a very stable differentiation reference independent of the differentiation stimulus.	Zhou et al., 2010	

*Further information on a selection of proteins with versatile known roles in the regulation of neuronal functioning and neurogenic processes found to be differentially expressed in the same manner in NGF and ns-Zr15, compared to PLL*.

The comparison of ns-Zr15_vs_PLL (Table S3) with ns-Zr15_vs_flat-Zr (Schulte et al., 2016a) shows that the impact of ns-Zr15 on the protein expression profile is very similar (24 proteins altered in the same manner; marked X in Table S3) with respect to the two flat surfaces (flat-Zr and PLL).

Pointing instead more specifically toward the differences between ns-Zr15 and NGF (ns-Zr15_vs_NGF, Table S2), the comparison reveals that 19 proteins (37%) are involved in cell proliferation and differentiation, 11 (22%) are receptors or players in signal transduction processes and 4 (8%) are related to Ca^2+^ signaling. Moreover, the ClueGo analysis highlights that these proteins are mainly involved in neurofilament formation and assembly (e.g., vimentin, an intermediate filament protein needed during initiation of neuritogenesis; Boyne et al., 1996) and some in protection against oxidative damage (Figure S3D).

For some of these proteins [including also proteins appearing only in ns-Zr15_vs_PLL (Table S3) and not in NGF_vs_PLL (Table S1), or expressed exclusively in ns-Zr15 (Table S5)] crucial and versatile functions in (post-)transcriptional and epigenetic regulation have been observed [e.g., DDB1 (Cang et al., 2006), Nedd4 (Drinjakovic et al., 2010; Wiszniak et al., 2013; Hsia et al., 2014); Dpy30 (Jiang et al., 2011); Nsun2 (Blanco et al., 2014; Hussain and Bashir, 2015), HMGB2 (Abraham et al., 2013), hnRNP A1 (Han et al., 2005; Li et al., 2007), Vbp1/prefoldin 3 (Millán-Zambrano and Chávez, 2014); details in Table 2: Differences–Neuronal differentiation/function]. Moreover, various proteins are of particular interest regarding a potential connection of mechanotransductive signaling and neuronal differentiation processes in the nanotopography-induced setting [e.g., Fat4 (Katoh, 2012; Zakaria et al., 2014; Ito et al., 2015), Versican (Wu et al., 2004; Paszek et al., 2014), Thrombospondin (Goicoechea et al., 2000; Adams et al., 2001; Barker et al., 2004; Xu et al., 2010; Paszek et al., 2014), ADAM12 (Kawaguchi et al., 2003; Eckert et al., 2017), Talin (Tan et al., 2015; Schulte et al., 2016c), NCoa2 (Voegel et al., 1996; Wyszynski et al., 2002; Spangler and Hoogenraad, 2007; Essmann et al., 2008; Asperti et al., 2009, 2010; Selak et al., 2009; Dasgupta et al., 2014; Geiger et al., 2014; Heisler et al., 2014), Ran(bp3/GAP) (Yudin and Fainzilber, 2009); marked in gray in Tables S2, S5; details in Table 2: Differences–IAC/Mechanotransduction]. This information is additionally validated by the IPA bioinformatics analysis of the proteins differentially expressed in ns-Zr15_vs_NGF (Table S2). This evaluation detected relevant protein networks modulated by the surface nanotopography related to: cell morphology, cellular assembly and organization, cellular movement, molecular transport, cell signaling, vitamin and mineral metabolism, cancer and invasion (Table 3).

**Table 2:** Differences between biochemically and mechanotransductively promoted neuronal differentiation at the protein level by comparing the conditions ns-Zr15, NGF, and PLL.

**Differences**	**Associated to** **Similarities and differences between biochemically and mechanotransductively promoted neuronal differentiation at the protein level**			
Examples of differentially expressed proteins comparing ns-Zr15 with NGF (Tables S1–S5) and their reported roles in neuronal differentiation/function and IAC/mechanotransduction				
**Protein name**	**ns-Zr15**	**Reported protein functions**	**References**	**Categories (Figure 2)**
**NEURONAL DIFFERENTIATION/FUNCTION**				
				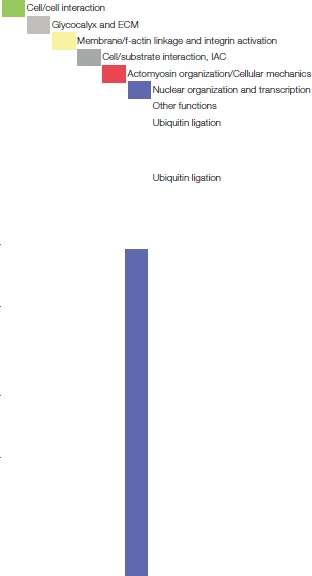
			
			
			
			
DDB1 (DNA damage-binding protein 1)	up	DDB1 and Nedd4 are both involved in ubiquitin ligation and are essential for the regulation of neuronal cell survival, axon branching and the maintenance of stem cell properties of neural crest cells. Nedd4 has many substrates with important function in neurons (e.g. ion channels, receptors). The mRNA expression of Nedd4 itself is regulated downstream of the PTEN/mTORC1 pathway.	Cang et al., 2006	
Nedd4 (neuronal precursor cell expressed and developmentally downregulated protein 4)	down		Drinjakovic et al., 2010; Wiszniak et al., 2013; Hsia et al., 2014	
Dpy30 (histone methyltrasferase complex regulatory subunit)	up	Dpy30, with its role in histone methylation, has been found to control embryonic stem cell fate specification, in particular toward the neural lineage.	Jiang et al., 2011	
Nsun2 (NOP2/sun domain family, member 2)	up	Mutations in this cytosine-5 RNA methyltransferase can lead to microcephaly (Dubowitz syndrome) and other neurodevelopmental disorders. Nsun2 can be found in neuronal dendrites and due to its mRNA methylation activity it is involved in the epigenetic control of postsynaptic functions and plasticity.	Blanco et al., 2014; Hussain and Bashir, 2015	
HMGB2 (high mobility group B2)	down	HMGB2 belongs to the family of chromatin structural proteins and is important in the control of neural stem cell proliferation and the regulation of neurogenesis and neuronal differentiation.	Abraham et al., 2013	
hnRNP A1 (heterogeneous nuclear ribonucleoprotein A1)	down	hnRNP A1 is known to be involved in alternative pre-mRNA splicing, in the neuronal setting as a silencing factor.	Han et al., 2005; Li et al., 2007	
Vpb1 (Von Hippel-Lindau binding protein 1)/prefoldin 3	down	The best known function of Vbp1/prefoldin 3 is its involvement, as a chaperone, in the folding processes of actin and tubulin monomers. Furthermore, it has been reported to participate in the regulation of the RNA polymerase II, chromatin dynamics and transcription.	Millán-Zambrano and Chávez, 2014	
**IAC/MECHANOTRANSDUCTION**				
Fat4 (protocadherin 4)	up	A hippo pathway-mediated role in neuronal migration and polarization for this protein has been reported recently. Interestingly and in line with our results, the Fat4 expression is controlled in a src-dependent manner by actin dynamics. The FAT4 protein is also thought to function upstream of YAP as a tumor suppressor, keeping cells from growing and dividing too rapidly or in an uncontrolled way. Further information on this protein regarding its role in calcium signaling-related processes can be found in the section Calcium Signaling/Homeostasis-Related Proteins Affected by the Cell/Nanotopography Interaction.	Katoh, 2012; Zakaria et al., 2014; Ito et al., 2015	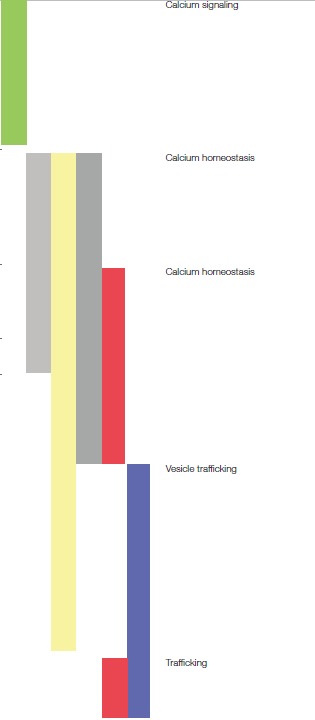
Vcan (versican)	up	Versican, a proteoglycan, promotes the outgrowth of neurites in NSC, hippocampal neurons and PC12. These effects were shown to be β1 integrin- and Erk-dependent. Proteins of the glycocalyx, such as thrombospondin 1 and versican are of particular interest regarding mechanosensing and -transduction, because the glycocalyx (especially bulky glycoproteins) has been recently found to have an important impact on integrin clustering and integrin-mediated signaling.	Wu et al., 2004; Paszek et al., 2014	
Thbs1 (thrombospondin 1)	up	This adhesive extracellular matrix glycoprotein has been found to influence focal adhesion disassembly and cytoskeletal organization in a fascin-mediated manner. In hippocampal neurons it can enhance synaptogenesis.	Goicoechea et al., 2000; Adams et al., 2001; Barker et al., 2004; Xu et al., 2010; Paszek et al., 2014	
ADAM12 (A disintegrin and metalloprotease 12)	down	ADAM12 has been reported to be capable of regulating β1 integrin, the actin cytoskeleton and focal adhesion turnover.	Kawaguchi et al., 2003; Eckert et al., 2017	
Talin	up	Talin is a crucial protein in integrin activation and the initial phase of IAC formation, providing the first link between integrins and f-actin. It is therefore essential for the build-up of tension in lamellipodia. In a neuronal context, activated talin is necessary in the growth cones and enhances neuritogenesis.	Tan et al., 2015; Schulte et al., 2016c	
NCoa2 (nuclear receptor coactivator 2)/GRIP1/TIF-2	up	NCoA2 represents on the one hand a transcriptional coregulator for various nuclear receptors (e.g., androgen, estrogen, glucocorticoid, steroid receptor) and facilitates the access for DNA transcription by histone acetylation. On the other hand it has been reported to be important for essential neuronal functions and dendrito- or synaptogenesis, in particular by coordinating neuronal and synaptic cargo and receptor (kainite, AMPA) trafficking. It can cooperate with other crucial proteins in these processes, such as SNAP25, 14-3-3 or liprin-α. The latter protein is furthermore known to be involved in the distribution and regulation of β1 integrin activation by stabilizing it in its inactive conformation at the cell membrane.	Voegel et al., 1996; Wyszynski et al., 2002; Spangler and Hoogenraad, 2007; Essmann et al., 2008; Asperti et al., 2009, 2010; Selak et al., 2009; Dasgupta et al., 2014; Geiger et al., 2014; Heisler et al., 2014	
Ran(bp3)RanGAP1	updown	The Ran GTPase (and its regulators) have versatile roles in neuronal cells, such as nucleocytoplasmic transport, trafficking, axon retrograde signaling and cytoskeletal dynamics.	Yudin and Fainzilber, 2009	

*Further information on a selection of proteins with prominent reported roles in the regulation of neuronal functioning and neurogenic processes and/or relevance for mechanotransduction differentially expressed comparing ns-Zr15 and NGF*.

**Table 3:** IPA bioinformatics analysis of the proteins differentially expressed in ns-Zr15_vs_NGF.

**Networks**	**Molecules in network**	**Score**
Cell Morphology, Cellular Assembly and Organization, Cellular Movement	Actin, AHNAK, Akt, Ap1, API5, caspase, CD3, Cg, CNIH4, Creb, ERK, estrogen receptor, FSH, GOT1, Gsk3, GZMB, HDLBP, Histone h3, HN1, IDH2, Lh, MAP2K1/2, MCAM, NCOA2, NGF, p85 (pik3r), PARP, Pkc(s), PLC gamma, RANGAP1, ROCK2, TMSB10/TMSB4X, Vegf, VGF, VIM	34
Cancer, Neurological Disease, Organismal Injury and Abnormalities	26sProteasome, ACADL, ALAD, CD163, CFH, DDB1, DYNC1H1, EML2, HISTONE, Histone h4, IgG, IL1, IL12 (complex), IL12 (family), Immunoglobulin, ING3, Insulin, Jnk, NEDD4L, NFkB (complex), Nr1h, P38 MAPK, PI3K (complex), Pka, PSMA1, PSMD11, Ras, Ras homolog, RNA polymerase II, RPA2, RPL6, RPL15,Tnf (family), TUBB2A,TXNL1	34
Cancer, Organismal Injury and Abnormalities, Reproductive System Disease	ACY1, Alpha Actinin, collagen, Collagen Alpha1, Collagen type I, Collagen type IV, Collagen(s), CTGF, ERK1/2, F10, F Actin, Focal adhesion kinase, HTRA1, INHBA, Integrin, Laminin, LDL, Mek, Mmp, Myosin, NEB, Pdgf (complex), PDGF BB, RANBP3, Rock, SELP, SERPINB8, Smad, Smad2/3, Sphk, TAGLN, Tgf beta, THBS1, TLN1, VCAN	26
Molecular Transport, Cell Signaling, Vitamin and Mineral Metabolism	12-hydroxyeicosatetraenoic acid, ADGRB2, AMT, ATP6V1A, C1QBP, Ca2+, CHAT, CNBP, CYP2D6, DLD, EGF, FAM136A, FFAR4, GCSH, GPX3, growth factor receptor, Hmgb2 (includes others), HNF4A, HTT, ILK, MGST3, MYC, Nefm, Neurotrophin, Ntrk1 dimer, PNPLA6, potassium channel, quinolinic acid, S1PR2, SCG2,SHC1, SLC24A3, sn-glycero-3-phosphocholine, TUBAL3, VGF	23
Cell Cycle, Cellular Development, Hair and Skin Development and Function	12-hydroxyeicosatetraenoic acid, ADAM12, CARS, CCDC80, CDK4, CDKN2A, CHRNB4, CTNND2, DPY30, DUSP4, E2F4, EIF5A, KIF3C, KRAS, LIFR, Mapk, mir-1260a, miR-1913 (and other miRNAs w/seed CUGCCCC), miR-378a-3p (and other miRNAs w/seed CUGGACU), neuroprotectin D1, NPM1, PRKCSH, Rac, RALB, Rho gdi, ROR1, RPS15, S100A12, S1PR2, SH3RF1, STMN2, TGFB1, TNF, TRIO, VTA1	14
Cardiovascular Disease, Connective Tissue Disorders, Dermatological Diseases and Conditions	LBR, Olfr1320	2

*Proteins differentially expressed in ns-Zr15_vs_NGF, either up- or downregulated or only expressed in the two conditions, were analyzed by IPA to identify possible networks*.

### Influence of the surface nanotopography roughness on the protein expression

#### Focus on ns-Zr15, ns-Zr25, NGF, PLL

As shown in Figure 1, the higher surface roughness R_q_ 25 nm RMS (ns-Zr25) also induced neuritogenesis in PC12 but to a lower degree (Schulte et al., 2016a). The rationale of this phenomenon is not completely clear yet. Therefore, in addition to the mentioned ns-Zr15, we have included ns-Zr25 into this proteomic approach (Tables S7–S12). The Venn diagram and work flow for the comparison of NGF, ns-Zr15, ns-Zr25 are shown in Figures S4A,B, respectively.

Several proteins (7 out of 26; marked in light gray in Table S8) found to be upregulated in ns-Zr25_vs_NGF (Table S8) are also upregulated in the comparison ns-Zr15_vs_NGF (Table S2) or ns-Zr15_vs_PLL (Table S3), suggesting that the biological processes triggered by the cell/nanostructure interaction are partially similar, even if the roughness parameter is increasing.

The proteins upregulated only in ns-Zr25_vs_NGF (Tables S8, S12), and not in the other conditions, are e.g., stress-induced proteins (such as CASC5, GPX3, A1M, and HSP90) and proteins involved in transport and trafficking. The data further demonstrates that the interaction of PC12 cells with higher roughness is accompanied by an increase of proteins related to formation/degradation of atherosclerosis plaques (APOB, SERPIND1), secretion, anti-inflammation activity and stress response (HMOX1, LOC681468, TXN, HMGN1, Cybasc3) while others are directly involved in gene expression control. Accordingly, the enrichment analysis of GO biological processes carried out by Panther on the proteins upregulated or only expressed in ns-Zr25 (Table 4) shows that the roughness increase triggers the expression of proteins involved in response to oxygen-containing compounds.

**Table 4:** Comparison of ns-Zr15_vs_ns-Zr25 with respect to biological processes.

**GO biological process complete**	**Fold enrichment**	***p*-value**
Regulation of peptidase activity (GO:0052547)	11.55	1.68E-02
Response to oxygen-containing compound (GO:1901700)	4.82	2.37E-02
Regulation of biological quality (GO:0065008)	3.29	4.24E-02
Negative regulation of cellular process (GO:0048523)	3.02	2.48E-02
Negative regulation of biological process (GO:0048519)	2.96	1.40E-02

*Gene Ontology Biological Process enrichment analysis by Panther on the proteins upregulated and expressed only in ns-Zr25*.

The proteome analysis of cells grown on ns-Zr25 also displays an increased expression of proteins involved in regulation of cell proliferation, differentiation and apoptosis, adhesion and trafficking, as well as intercellular signaling pathways. Some of these proteins indicate a strong neuronal differentiation-promotive effect also for this substrate [e.g., syntaxin 4 (Kennedy et al., 2010; Mohanasundaram and Shanmugam, 2010), clathrin (Heuser and Reese, 1973; Cosker and Segal, 2014), HMGN1 (Deng et al., 2013; Nagao et al., 2014), and SCN1B (Namadurai et al., 2015), details in Table 5], consistent with our results in primary hippocampal neurons where ns-Zr25 had the most significant effect on neuron differentiation and maturation (Schulte et al., 2016b). However, the induction of many stress-related proteins suggests that the substrate situation is becoming suboptimal for PC12 cells leading to the altered expression of proteins that are essential for the regulation of neuronal survival [e.g., CREM (Mantamadiotis et al., 2002; Wu et al., 2012), NPM1 (Qing et al., 2008; Pfister and D'Mello, 2015); details in Table 5].

**Table 5:** Comparison of ns-Zr25_vs_ns-Zr15 regarding the influence of the surface nanotopography roughness on the protein expression.

	**Associated to** **Influence of the surface nanotopography roughness on the protein expression**			
**Protein name**	**ns-Zr15**	**Reported protein functions**	**References**	**Categories (Figure 2)**
				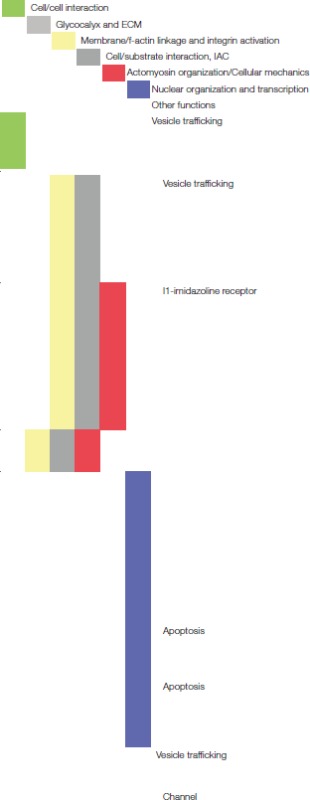
			
			
			
			
Rab14	up	Rab14 is involved in endocytic recycling and regulates therewith ADAM-dependent N-Cadherin shedding affecting cell-cell adherens junctions, cell polarization and migration.	Linford et al., 2012	
CLTC (clathrin heavy chain 1)	down	Clathrin is long-known to play a crucial role in neuronal signaling by mediating synaptic vesicle endocytosis, receptor retrieval and retrograde transport regulating neuronal functions (e.g., neuron survival, axon growth and guidance). Apart from this role in neuronal signaling by mediating endocytosis of synaptic receptors, clathrin controls also focal adhesion dimensions in migrating cells by regulating the endocytosis of inactive integrins.	Heuser and Reese, 1973; Ezratty et al., 2009; Cosker and Segal, 2014	
Nischarin	up	Nischarin is associated with versatile and complex processes. It has been reported to negatively regulate cell migration but to foster neurite outgrowth, also in PC12 cells. It binds non-ligated integrins (e.g., α5β1), acting as a sort of integrin inhibitor, and modulates the LIMK/cofilin pathway and PAK/Rac1 activation affecting thereby the actin dynamics. In addition, in differentiated PC12 nischarin behaves as I1-imidazoline receptor and activates protein kinase C (PKC), extracellular signal-regulated kinase (ERK) and c-jun N-terminal kinase (JNK) promoting the neuronal differentiation and survival.	Alahari et al., 2000, 2004; Zhang and Abdel-Rahman, 2006; Ding et al., 2008, 2015; Pouwels et al., 2012	
ArhGEF1/P115-RhoGEF	up	ArhGEF1 interacts with Gα13 and regulates RhoGTPase activity and controls focal adhesion and stress fiber formation in response to fibronectin.	Hart et al., 1998; Kozasa et al., 1998; Dubash et al., 2007	
HMGN1 (high mobility group nucleosome binding domain 1)	down	HGMN1, a nucleosome-binding protein, is important for the structural organization of the chromatin and fine-tunes transcription profiles along the neuronal lineage. The loss of this protein lowers the amount of neural progenitor cells in the brain, whereas increased expression fosters astrogenesis instead of neurogenesis.	Deng et al., 2013; Nagao et al., 2014	
SF3B2 and 5 (splicing factor 3B subunit 2 and 5)	up	SF3B2 and 5 belong to the U2 snRNP spliceosome complex and is therefore involved in the splicing pre-mRNA. Dysfunction of this complex leads to a general perturbation of alternative splicing causing neurodegeneration.	Jia et al., 2012	
CREM (cAMP-responsive element modulator)		CREM is a member of the cAMP-responsive element binding protein family and important in the transcriptional regulation. It is upregulated as response to nerve tissue injury and repair, probably as a pro-apoptotic factor.	Mantamadiotis et al., 2002; Wu et al., 2012	
NPM1 (nucleolar phosphoprotein B23, numatrin)/nucleophosmin/ B23	down	NPM1 is a nucleolar protein with versatile function, especially also in a neuronal context where it is involved in the control of growth, differentiation and apoptosis. Expressed in levels higher than the physiological ones, it can cause neuronal death.	Qing et al., 2008; Pfister and D'Mello, 2015	
Stx4 (syntaxin 4)	down	Syntaxin 4 is an essential postsynaptic t-SNARE protein involved in exocytosis and synaptic plasticity.	Kennedy et al., 2010; Mohanasundaram and Shanmugam, 2010	
SCN1B (sodium voltage-gated channel beta subunit 1)	down	SCN1B belongs to the NaV channel family which is important in the initiation of action potentials. The beta subunits are associated to the pore-forming alpha subunits and fine-tune the channels' excitability.	Namadurai et al., 2015	

*Selected proteins with reported roles in neuronal survival, functioning, differentiation and/or mechanotransduction that showed differential expression in the ns-Zr15_vs_ns-Zr25 comparison*.

Compared to ns-Zr15, in ns-Zr25 (Table S7) there was decreased protein expression of tumor suppressors involved in apoptosis (PARK7, GZMB, SRSF1, FAT4) and cytoskeletal proteins that play essential roles in the integrin signaling. The IPA confirms the latter observation by identifying ILK (integrin-linked kinase) signaling as the only canonical pathway significantly decreased on ns-Zr25 (Z score-1, proteins CDH1, FN1, ACTN4, TMSB10/TMSB4X) (Table 6). Intriguingly, this pathway has been reported to be pivotal in the regulation of IAC architecture/composition and to be sensitive to integrin ligand density of the substrate (Elad et al., 2013). In the context of mechanosensing, lysophosphatidylcholine acyltransferase (Lpcat2b) expression only in the ns-Zr25 condition is intriguing (Table S12). This protein converts lysophosphatidylcholine in phosphatidylcholine; a process essential in the regulation of membrane dynamics (i.e., curvature/bending, tension), recruitment of F-BAR proteins and membrane/f-actin linkage (Anitei and Hoflack, 2012). Also regarding cell/cell contact, IAC and actomyosin organization some changes are noteworthy [such as Rab14 (Linford et al., 2012), clathrin (Ezratty et al., 2009), nischarin (Alahari et al., 2000, 2004; Zhang and Abdel-Rahman, 2006; Ding et al., 2008, 2015; Pouwels et al., 2012), ArhGEF1/P115-RhoGEF (Hart et al., 1998; Kozasa et al., 1998; Dubash et al., 2007); details in Table 5]. In addition, SF3B2 and 5 (upregulated in the cells on ns-Zr15, Table S11), are components of the spliceosomal U2 small nuclear ribonucleoprotein particle that has an important role in neuronal transcriptional regulation (Jia et al., 2012) (details in Table 5).

**Table 6:** IPA bioinformatics comparison of ns-Zr15_vs_ns-Zr25.

**Ingenuity canonical pathways**	***p*-Value**	**z-score**	**Molecules**
Epithelial Adherens Junction Signaling	5,37E-03	NaN	CDH1, ACTN4, TUBB
Germ Cell-Sertoli Cell Junction Signaling	8,71E-03	NaN	CDH1, ACTN4, TUBB
Sertoli Cell-Sertoli Cell Junction Signaling	1,02E-02	NaN	CDH1, ACTN4, TUBB
GIalpha12/13 Signaling	3,98E-02	NaN	CDH1, ARHGEF1
ILK Signaling	1,32E-03	−1	CDH1, FN1, ACTN4, TMSB10/TMSB4X
Actin Cytoskeleton Signaling	2,34E-03	0	FN1, ARHGEF1, ACTN4, TMSB10/TMSB4X
Heme Degradation	3,09E-04	NaN	HMOX1, BLVRB
IL-10 Signaling	1,29E-02	NaN	HMOX1, BLVRB
Unfolded protein response	7,41E-03	NaN	P4HB, EIF2AK3
Acute Phase Response Signaling	7,59E-04	NaN	PLG, HMOX1, FN1, SERPIND1
Coagulation System	3,24E-03	NaN	PLG, SERPIND1
Insulin Receptor Signaling	4,90E-02	NaN	PTPN1, STX4

*Proteins expressed in a differential manner in the comparion ns-Zr15_vs_ns-Zr25 were analyzed by IPA to identify canonical pathways affected by the different nanotopography characteristics*.

In conclusion, it emerges that several critical proteins for membrane dynamics and configuration, integrin activation, IAC assembly and linkage to the f-actin are affected even by relatively subtle differences in the nanotopographical characteristics (Schulte et al., 2016a). This impact suggests a prominent role of the mentioned proteins in mechanosensing of topographical surface features.

### Impact of the cellular interaction with the neuritogenesis-inducing cluster-assembled zirconia surface on protein phosphorylation

#### Phosphoproteomic data of ns-Zr15, ns-Zr25, NGF, PLL, flat-Zr

The proteomic data presented here are complemented by an analysis of the phosphorylation state of proteins in the outlined experimental conditions, providing more profound information on the signaling pathways and potential specific key mediators (Tables S13–S22). A detailed analysis of individually identified phosphorylated proteins with interesting functions in the framework of this study is displayed in Table 7.

**Table 7:** Comparison of the conditions ns-Zr15, ns-Zr25, NGF, PLL and flat-Zr to analyse the impact of the cellular interaction with the neuritogenesis-inducing cluster-assembled zirconia surface on protein phosphorylation.

**Associated to** **Impact of the cellular interaction with the neuritogenesis-inducing cluster-assembled zirconia surface on protein phosphorylation**			
**Protein name**	**Reported protein functions**	**References**	**Categories (Figure** 2**)**
			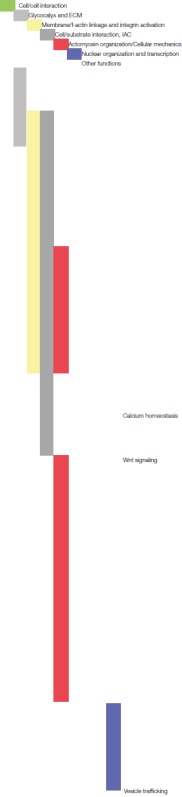
		
		
		
		
Nidogen-1	The basement membrane protein nidogen-1 is known to be important in the regulation of hippocampal synaptic plasticity and network excitability.	Vasudevan et al., 2010	
Brorin	Brorin has been reported to favor neurogenesis and to inhibit astrogenesis contributing to axon guidance in the zebrafish forebrain.	Miyake et al., 2017	
Galectin-8	Galectin-8 is a secreted ECM protein and matricellular modulator of cell adhesion that is bound by integrins which regulates cell adhesion and survival, promoting or inhibiting, dependent on whether it is present in a soluble or immobilized manner.	Hadari et al., 2000; Zick et al., 2004	
Ptprf (receptor-type tyrosine-protein phosphatase F)/LAR (leukocyte common antigen-related)	The Ptprf/LAR receptor, a neuronal adhesion molecule essential in synapse maturation, is particularly interesting with respect to IAC, mechanotransduction and calcium signaling. The presence of this receptor in focal adhesions (FA) is controlled in a negative manner by myosin II-generated force and has been shown to have the capacity to regulate FAs (in mouse embryonic fibroblasts). It interacts in a CaMKII (Ca^2+^/calmodulin-dependent protein kinase II)-regulated way with liprin-α 1. The liprin-α 1/LAR interaction determines LAR distribution and therefore synapse morphogenesis. Moreover, Ptprf/LAR can be found, to a minor extent, tyrosine-phosphorylated in the adult brain. The function of this phosphorylation is yet unknown but could be important for the binding of SH2/SH3 domain-containing adaptor proteins.	den Hertog et al., 1994; Johnson and Van Vactor, 2003; Dunah et al., 2005; Kuo et al., 2011; Um and Ko, 2013; Sarhan et al., 2016	
Gpr56 (G protein-coupled receptor 56)	Malfunctions of Gpr56 can cause the neurodevelopmental disease polymicrogyria. In the brain it is predominantly expressed in neuronal progenitor cells (NPC) in regions of postnatal neurogenesis where it is involved in the control of brain convolution/patterning in the cerebral cortex in an integrin α3β1-dependent manner. Moreover, Gpr56 operates together with Gα13 in the Rho-mediated regulation of NPC adhesion/migration. Gα13 again is essential in integrin signaling.	Piao et al., 2004; Iguchi et al., 2008; Gong et al., 2010; Shen et al., 2012; Jeong et al., 2013; Bae et al., 2014	
ROCK (Rho-associated, coiled-coil-containing protein kinase)	ROCK/RhoA activity has a complex role in neuritogenesis. Although on the one hand it is known to be inhibitory for neuritogenesis, and in particular for the initial neurite formation, on the other hand spatially restricted ROCK/RhoA activity is also essential to suppress lamellipodial protrusions, thereby consolidating neurites/axons by maintaining the growth cone polarity. ROCK and its RhoA binding activity is tightly regulated by phosphorylation downstream of src and contributes to the modulation of focal adhesion turnover.	Yamaguchi et al., 2001; Loudon et al., 2006; Lee et al., 2010; Schulte et al., 2010	
GFRA1 (GDNF family receptor α-1)	GFRA1 was found to form a complex with β1 integrin, together with Ret and NCAM-140, and to play an important role in the differentiation of neurons in the olfactory system and the survival of glutamatergic cortical neurons.	Cao et al., 2008; Marks et al., 2012; Konishi et al., 2014	
G3BP1 (Ras GTPase-activating protein-binding protein 1)	G3BP1 has been reported to have an impact on neuronal sprouting by promoting the formation of tau mRNA ribonucleoprotein granules and can be found associated with α5β1 integrin-containing complexes. Moreover, G3BP1 deficiency impairs the synaptic plasticity and calcium homeostasis in hippocampal neurons.	Meng et al., 2004; Martin et al., 2013; Moschner et al., 2014	
ArhGAP18 (Rho GTPase activating protein 18)	ArhGAP18 has been shown to be involved, as negative regulator, in the control of RhoA activity and stress fiber formation by increasing the GTPase activity of Rho and stabilizing the RhoA-GDP inactive form. The protein, interacting with RhoA, has been described recently as YAP effector in the actomyosin-dependent regulation of tissue tension. A specific role of this protein in neurons has not been reported so far, but its expression level decreases in neurospheres during differentiation and it appeared as a gene associated with schizophrenia in a screening for single nucleotide polymorphisms. For another ArhGAP family member, ArhGAP15, a contribution in the neurogenesis of hippocampal neurons has been shown very recently.	Gurok et al., 2004; Potkin et al., 2009; Maeda et al., 2011; Porazinski et al., 2015; Zamboni et al., 2016	
ASPM (Abnormal spindle-like microcephaly-associated protein)	ASPM is known to contribute to the regulation of neuronal differentiation processes by actomyosin-dependent actions. As the protein name indicates, this protein is involved in the control of brain size and mutations of this protein can be responsible for developing the neural disorder microcephaly. ASPM is a positive regulator for Wnt signaling and its expression is essential for accurate neurogenesis. Furthermore, recently it has been found that the drosophila ortholog of this protein interacts with and regulates myosin II localization, thereby controlling neuroepithelium morphogenesis by mechanobiological events.	Buchman et al., 2011; Rujano et al., 2013	
Cofilin/destrin/ADF (actin depolymerising factor)	Cofilin is essential for actin cytoskeletal organization by regulating the severing of f-actin and the turnover rate of actin and therewith, in the neuronal context, the actin retrograde flow in neurite growth cones of the developing brain. Phosphorylation negatively regulates its actin binding and thereby controls the f-actin homeostasis.	Hawkins et al., 1993; Jovceva et al., 2007; Flynn et al., 2012	
Septin-2	Septin-2 modulates actomyosin contractility by binding myosin II and recruiting regulatory proteins. Septin phosphorylation controls the assembly of septins into highly ordered polymers. Interestingly, septin-2 has been found to be phosphorylated in post-mitotic neurons.	Spiliotis and Nelson, 2006; Joo et al., 2007	
KMT2D (histone-lysine N-methyltransferase 2D)/MLL4 (mixed-lineage leukemia 4)	This protein is a mammalian histone H3 lysine 4 (H3K4) mono-methyltransferase essential in differentiation-specific gene activation. It has been shown to participate in the regulation of neuronal differentiation, facilitating the activation of differentiation-specific genes (e.g., nestin).	Dhar et al., 2012	
RTCB (RNA 2′,3′-cyclic phosphate and 5′-OH ligase)	This RtcB RNA ligase participates in tRNA ligation and it is involved in the regulation of neuronal growth and axon regeneration.	Kosmaczewski et al., 2015	
E2F4	This transcription factor has been shown to play a role in neuronal differentiation and neuritogenesis.	Persengiev et al., 1999	
Rab23 (Ras-related protein 23)	Rab23 participates to endocytic vesicle trafficking and is involved in the regulation of sonic hedgehog signaling in neural tube patterning.	Eggenschwiler et al., 2001; Evans et al., 2003	

*Selected proteins found to be differentially phosphorylated that have essential known functions with respect to processes relevant for mechanotransductive signaling and/or neuronal functioning and differentiation*.

Specifically, the phosphoproteomic data shows that the cell/nanotopography interaction (ns-Zr15) leads to a differential phosphorylation of various proteins reported to be important in controlling IAC dimension/composition, the actin cytoskeleton, and the cellular mechanics [e.g., ADAM12 (Kawaguchi et al., 2003; Eckert et al., 2017); Table 2, nidogen-1 (Vasudevan et al., 2010), brorin (Miyake et al., 2017), Ptprf/LAR (den Hertog et al., 1994; Johnson and Van Vactor, 2003; Dunah et al., 2005; Kuo et al., 2011; Um and Ko, 2013; Sarhan et al., 2016), Gpr56 (Piao et al., 2004; Iguchi et al., 2008; Gong et al., 2010; Shen et al., 2012; Jeong et al., 2013; Bae et al., 2014), ROCK (Yamaguchi et al., 2001; Loudon et al., 2006; Lee et al., 2010; Schulte et al., 2010), GFRA1 (Cao et al., 2008; Marks et al., 2012; Konishi et al., 2014), G3BP1 (Meng et al., 2004; Martin et al., 2013; Moschner et al., 2014), ArhGAP18 (Gurok et al., 2004; Potkin et al., 2009; Maeda et al., 2011; Porazinski et al., 2015; Zamboni et al., 2016), ASPM (Buchman et al., 2011; Rujano et al., 2013), cofilin (Hawkins et al., 1993; Jovceva et al., 2007; Flynn et al., 2012), septin-2 (Spiliotis and Nelson, 2006; Joo et al., 2007); details in Table 7]. Furthermore, several proteins essential in epigenetic and (post-)transcriptional regulation of gene expression are modulated at the phosphorylation level [e.g., KMT2D (Dhar et al., 2012), RtcB (Kosmaczewski et al., 2015), E2F4 (Persengiev et al., 1999); details in Table 7]. Regarding this latter aspect, it is noteworthy that lipin-1 phosphorylation is affected by the interaction with ns-Zr15. This phosphatidic acid phosphatase is important in lipid synthesis and SREBP-mediated transcriptional regulation (e.g., Fasn expression, see Table 1). Its phosphorylation is regulated by mTOR which thereby also controls its intracellular localisation and the lamin A-dependent nuclear organization (Peterson et al., 2011; Eaton et al., 2013).

**Figure 2:**
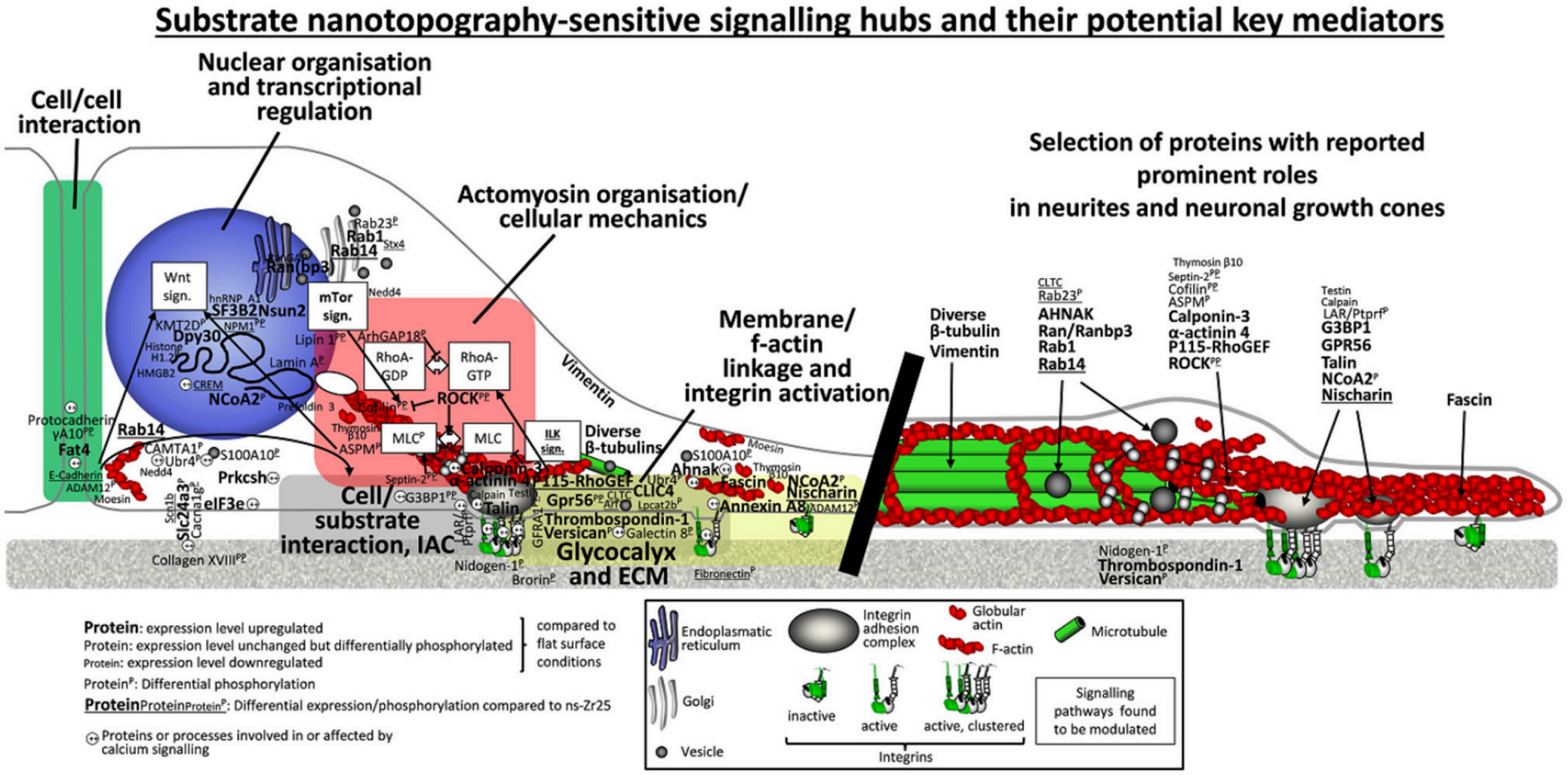
Schematic representation of the potential relation and crosstalk among different signaling pathways modulated by the neuron/nanotopography interaction. On the left the illustration arranges proteins dissected from the whole (phospho)proteomic data set [i.e., proteins with altered expression and/or phosphorylation level in the indicated conditions, including also some proteins from Schulte et al. (2016a)] into several categories and sets them into a (potential) relation to each other with respect to their reported cell biological function and context. The main categories are visualized as follows; cell/cell interaction (green box), glycocalyx and ECM (box with gray patterned filling), cell substrate interaction and IAC (gray box, gray oval represents the IAC), membrane/f-actin linkage and integrin activation (yellow box), actomyosin organization/cellular mechanics (red box), nuclear organization, and transcriptional regulation (blue circle, representing the nucleus). Further information on the reported specific functions of the individual proteins, justifying their categorisation, can be found in the corresponding tables throughout the manuscript. Moreover, on the right a selection of proteins is listed with association to their functions in neuronal differentiation processes, particularly in neurite growth cones.

The alterations (regarding expression and phosphorylation levels) extend in a consistent manner our previous results (Schulte et al., 2016a), accentuating additionally the impact of the cell/nanotopography interaction on mechanotransductive processes and defining more precisely nanotopography-sensitive signaling hubs (Figure 2).

**Figure 3:**
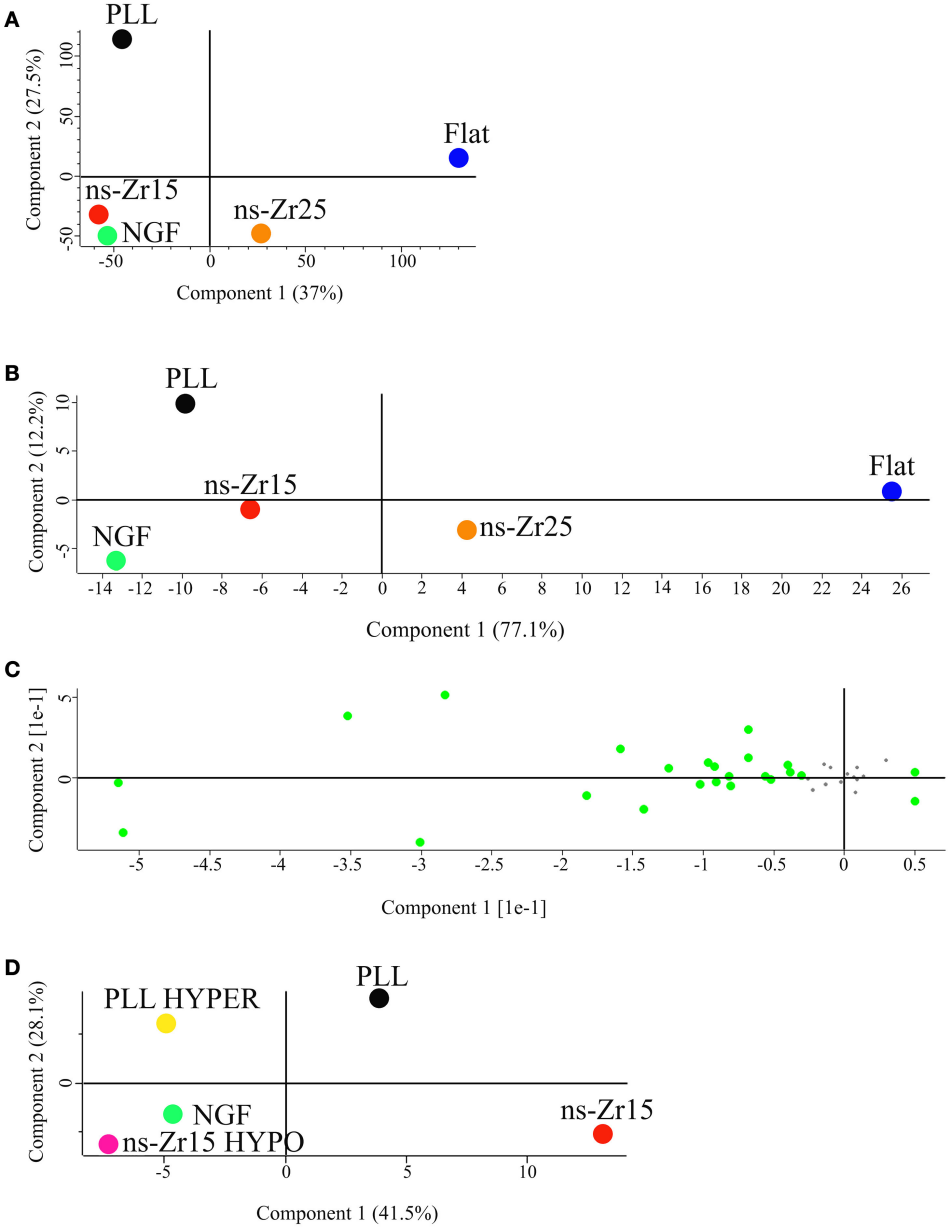
Principal component analysis (PCA) on the phosphoproteome of PC12 cells grown in different experimental conditions. **(A)** PCA analysis of the phosphopeptides of PC12 cells in the experimental conditions PLL, NGF, ns-Zr15, ns-Zr25, and flat-Zr. **(B)** PCA analysis of the sequence phospho-motifs present in the phosphoproteome data of PLL, NGF, ns-Zr15, ns-Zr25, and flat-Zr. **(C)** Visual representation of the PCA analysis of the sequence phospho-motifs. All the substrate motifs that are more relevant in the PCA analysis are marked in green. **(D)** PCA analysis of the phosphopeptides of PC12 cells in the experimental conditions PLL, NGF, ns-Zr15, PLL hyper, and ns-Zr15 hypo.

To find relevant patterns and specific differences in signaling processes related to the diverse conditions (PLL, NGF, ns-Zr15, ns-Zr25, and flat-Zr) a principal component analysis (PCA) was carried out on the corresponding phosphoproteomes. The analysis, applied to all the peptides found phosphorylated in these 5 conditions, reveals at a glance that the phosphoproteomes of NGF and ns-Zr15 cluster together (confirming again the common outcome of a differentiated cell). Flat-Zr and PLL instead are at the opposite ends of the plot (Figure 3A), suggesting that the cells on these two substrates behave very differently as far as protein phosphorylation concerns, in agreement with the other data reported so far.

If the same analysis is carried out focusing only on the sequence phospho-motifs present in the phosphoproteome data, a similar plot can be obtained (Figure 3B), but in this case a more evident separation can be observed between NGF and ns-Zr15, indicating that the kinases and phosphatases involved are, at least in part, different. Figure 3C reports in green all the substrate motifs that are more relevant in the PCA analysis, accounting for the 16% of all the phospho-motifs present in the phosphopeptides.

The enrichment analysis of these phospho-motifs, carried out by Panther and David, shows that there is a highly significant enrichment (*p* ≤ 0.05) of few signaling pathways in the cells on ns-Zr15 (Table 8). The angiogenesis and VEGF signaling pathways are in line with the processes mentioned throughout this work as they comprise many players also involved in focal adhesion, MAPK and Ca^2+^ signaling. In addition, the results suggest that the differences between PC12 cells grown on ns-Zr15 (compared to the NGF condition) could be partially ascribed to a modulation within the Wnt pathway (Table 8). An indicator is e.g., the downregulation of E-cadherin in the cells on ns-Zr15, considering the known crosstalk between (E-)cadherin cell adhesion and canonical Wnt signaling by release of β-catenin (Heuberger and Birchmeier, 2010). Interestingly, Wnt expression in PC12 cells leads to an upregulation of E-cadherin and a flat epithelial-like cell morphology associated with unresponsiveness to NGF-induced neuritogenesis (Bradley et al., 1993). In epithelial cells E-cadherin-mediated cell/cell adhesions are essential in mechanically connecting the intercellular actomyosin machineries to regulate tissue organization (Lecuit and Yap, 2015). Moreover, it has been demonstrated in human embryonic stem cells that the surface nanotopography has an impact on E-cadherin expression level (Chen et al., 2012). The potential impact of the substrate nanotopography on Wnt signaling in a neuronal setting is therefore an interesting issue for further investigations.

**Table 8:** Enrichment analysis of the kinases substrate motifs that are more relevant in the PCA analysis of phospho-sites differently expressed in ns-Zr15_vs_NGF.

	**Fold enrichment**	***p*-value**
**PANTHER PATHWAYS**		
Heterotrimeric G-protein signaling pathway-rod outer segment phototransduction	>100	1.47E-02
VEGF signaling pathway	78.4	4.40E-02
Parkinson disease	64.84	1.72E-03
CCKR signaling map	54.74	8.16E-05
Angiogenesis	45.05	5.07E-03
Wnt signaling pathway	22.45	3.96E-02
**DAVID PATHWAYS**		
Wnt signaling pathway	27.58865	1.93E-04
VEGF signaling pathway	48.625	1.198-03
GABAergic synapse	33.53448	2.503-03
Gap junction	33.15341	2.56-03
GnRH signaling pathway	31.71196	2.795-03
Inflammatory mediator regulation of TRP channels	25.36957	4.333-03
Thyroid hormone signaling pathway	25.36957	4.333-03
Tight junction	20.54577	6.542-03
Oxytocin signaling pathway	18.23438	8.247-03

*The ns-Zr15_vs_NGF analysis of the phospho-motifs was carried out by Panther and David (p ≤ 0.05)*.

In the comparison ns-Zr15_vs_ns-Zr25 (Tables S20–S22), apart from various already mentioned proteins, lamin A appeared as differentially phosphorylated. This protein is particularly interesting in the context of mechanotransduction representing one of the intermediate filaments that forms the interior of the nuclear envelope. It was found to be involved in the regulation of nuclear architecture/biophysics, chromatin organization, and transcription regulation at the end of mechanotransductive signaling cascades that influence differentiative processes (Swift et al., 2013). Its differential expression during adult neurogenesis proposes a potential role in it; however, to date details remain still unclear (Takamori et al., 2007). Further proteins found to be phosphorylated on ns-Zr25 are Galectin-8 (Hadari et al., 2000; Zick et al., 2004), and Rab23 (Eggenschwiler et al., 2001; Evans et al., 2003) which have essential reported functions in cell adhesion/survival and neuronal development, respectively (Table 7).

Altogether, many proteins that are important in neurogenic and/or mechanotransductive processes are differentially expressed and/or phosphorylated upon cellular interaction with the cluster-assembled zirconia surface that promotes neuritogenesis. Combining the analysis of our proteomic data with information available on these proteins and their functions, suggests a dynamic and complex modulation of an entire signaling network by the cell/nanotopography interaction that is in control of cellular behavior and fate, i.e., in this case neuronal differentiation. We were able to dissect potential nanotopography-sensitive key elements regulated within a mechanotransductive sequence, identifying many proteins that can be assigned to these principal categories: cell/cell adhesion, ECM and glycocalyx, cell/substrate interaction and IAC, integrin activation and membrane/f-actin linkage, integrin adhesion complexes, actomyosin organization/cellular mechanics and nuclear organization and transcriptional regulation (Figure 2).

### Alterations in cellular processes and signaling by the modulation of cellular tension

#### Focus on phosphoproteomic data of ns-Zr15, ns-Zr15 hypo, PLL hyper

In our previous paper we identified the alteration of the cellular nanomechanical properties as critical for the signal integration within the nanotopography-dependent mechanotransductive sequence that fostered neuronal differentiation. The interaction with the nanostructured surface dictated the IAC nanoarchitecture/dynamics and cytoskeletal organization in a manner that consequentially resulted in a softer membrane/cytoskeletal layer of the neuronal cell. Compensating this effect by a hypoosmotic gradient (causing cell swelling and an increase of cell tension) counteracted gradually the nanostructure-induced neuritogenesis on the morphological level (Schulte et al., 2016a).

**Figure 4:**
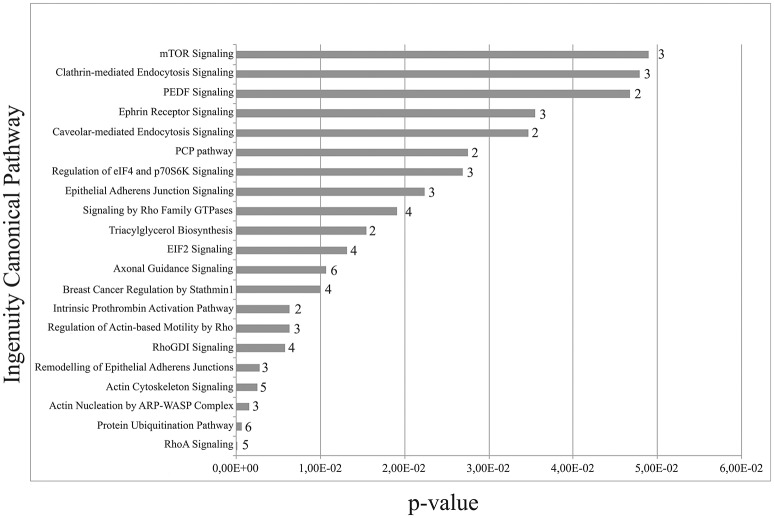
Comparison of ns-Zr15_vs_ns-Zr15 hypo. IPA canonical pathways enrichment analysis of ns-Zr15_vs_ns-Zr15 hypo.

The mechanotransduction dependency of the nanotopography-promoted differentiation was broadly validated by a proteomic comparison (Tables S23–S24) of PC12 cells interacting with the ns-Zr15 in the isoosmotic standard medium (ns-Zr15) or instead in the presence of hypoosmotic medium (ns-Zr15 hypo). Many proteins found to be expressed only in ns-Zr15 (Table S24) or to be downregulated in ns-Zr15 hypo (Table S23) can be classified as proteins involved in RhoGTPase-controlled cytoskeletal organization according to the IPA canonical pathways enrichment analysis (Figure 4). Consistent with the hypoosmotic manipulation of the membrane tension, clathrin- and caveolar-mediated endocytosis appeared among the five most affected pathways in this evaluation (Figure 4). These pathways are regulated by and respond to modulations of the membrane tension and are crucially involved in cell volume and shape control (Raucher and Sheetz, 1999; Sinha et al., 2011; Gauthier et al., 2012).

**Figure 5:**
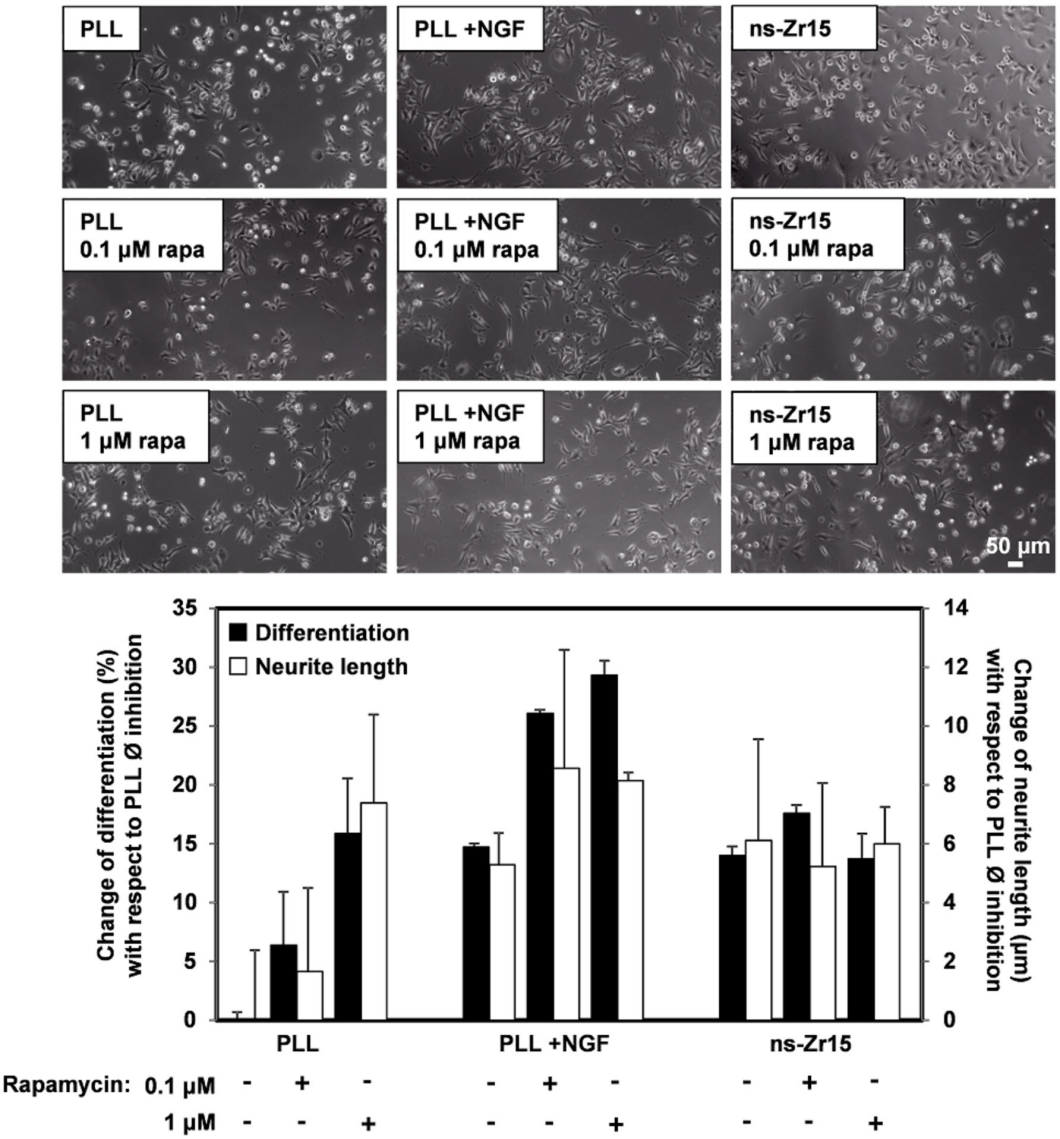
Effect of rapamycin inhibition on neurite outgrowth of PC12 cells on PLL and ns-Zr15. The phase contrast images and the graph display the reaction of PC12 cells on PLL (± NGF) or on ns-Zr15 after rapamycin treatment at two different concentrations: 0.1 and 1 μM. The graph summarizes the global statistics of two independent experiments showing the change of the differentiation rate and neurite length compared to the PLL –NGF Ø inhibition, with in total 367–943 cells and 216–661 neurites quantified for the differentiation rate (black bars), respectively neurite length (white bars). The bars show the average (mean ± s.d.) of the two experiments.

Intriguingly, mTOR signaling emerged as the strongest modulated pathway in this analysis (Figure 4). mTOR signaling represents a highly conserved pathway known to be an integrative master regulator of many cellular processes/pathways at the interface of intracellular and extracellular signals (Laplante and Sabatini, 2009), also in regard to neurogenic events (Garza-Lombó and Gonsebatt, 2016). Inhibition of mTOR(C1) with rapamycin had different effects on flat and nanostructured substrates. On PLL there was an increase of neurite outgrowth upon rapamycin inhibition both in the absence, or presence, of NGF. In the latter, the effect was additive to the NGF-induced increase, reproducing data reported by others (Parker et al., 2000). On ns-Zr15 instead no significant impact, neither promotive nor inhibitory, with respect to neurite outgrowth was observable (Figure 5). It can be speculated that the boosted neuritogenesis on PLL is due to an induction of mTORC2 activation triggered by the rapamycin-mediated inhibition of mTORC1 as a negative feedback between the two mTORCs is known (Xie and Proud, 2013). Moreover, mTORC2 has been shown to be involved in the regulation of actin dynamics and morphology of neurons in a Rac/PAK-dependent signaling pathway that controls cofilin phosphorylation (Huang et al., 2013; Thomanetz et al., 2013). Very recently, it has been demonstrated in DRG neurons that topographical features can potentiate mTORC2 guiding neurite outgrowth (Thomson et al., 2017). On ns-Zr15 the mTORC2 might already be induced by the cell/nanotopography interaction and thus rapamycin treatment does not further affect neurite outgrowth. The altered cofilin phosphorylation (Table 7) is in line with this (Huang et al., 2013). The varying impact of mTOR(C1) inhibition by rapamycin depending on whether the cells interact with a flat or a nanotopographical surface makes mTOR signaling an interesting and promising target for further studies in this context but goes beyond the scope of this work.

Moreover, prominent markers for (developing) neurons and neurite outgrowth [such as BASP1, MAP1B, or β-tubulin (TUBB5)] are strongly downregulated in the hypoosmotic condition. The same is true for many proteins involved in the actin polymerisation machinery and the cytoskeletal organization [(e.g., Capg, Arpc1b, 3 and 5, Capzb, fascin) which are crucial for the realization of neuritogenesis (da Silva and Dotti, 2002; Fletcher and Mullins, 2010); (Tables S23, S24)]. The alterations in the protein expression profile are largely mirror-inverted to those seen in the comparison ns-Zr15_vs_flat-Zr (Schulte et al., 2016a). 37 proteins have an opposite expression level in these two comparisons (marked **X** in Table S23), whereas only 5 proteins are altered in the same way (marked **x** in Table S23).

On the other hand, a hyperosmotic shock applied to cells on PLL-coated glass (resulting in a decrease of membrane tension) led morphologically to the outgrowth of neurites (Figure 1, Figure S1). The proteomic data (Table S25) disclosed that the neuritogenesis was accompanied by a modification of the protein profile similar to those found in ns-Zr15_vs_flat-Zr (Schulte et al., 2016a). 39 proteins had the same alteration of the expression level (marked in **x** in Table S25). However, proteins known to be involved in IAC (abundant e.g., in the ns-Zr15_vs_flat-Zr comparison; Schulte et al., 2016a) are basically missing here. In addition, 16 proteins also showed an opposite expression level modification (marked **X** in Table S25).

The PCA analysis carried out on the phosphopeptides differentially expressed in these conditions (ns-Zr15, ns-Zr15 hypo, NGF, PLL hyper, PLL) indicates that either hypoosmotic swelling on ns-Zr15, as well as the hyperosmotic shock on PLL, moves the profiles partially closer to the NGF condition. This emphasizes again that ns-Zr15 hypo basically lost its nanotopography-specific features, whereas PLL hyper has gained at least some characteristics of the NGF condition (Figure 3D).

Overall, these proteomic data further reinforce that the modulation of the cellular nanomechanical properties is a key integrating signal causally linked to the change of the cellular program and the differentiation processes that we discussed in our previous work (Schulte et al., 2016a), with a potential involvement of mTOR signaling constituents.

### Calcium signaling/homeostasis-related proteins affected by the cell/nanotopography interaction

#### Focus on proteins involved in calcium signaling and homeostasis

Alterations of the integrin/ECM interaction (e.g., in growth cone filopodia; Kuhn et al., 1998; Gomez et al., 2001; Gui et al., 2006) and cellular biomechanics can modulate another important mechanotransduction-susceptible pathway; that is calcium signaling regulated by Ca^2+^ influx passing mechanosensitive membrane channels (Vogel and Sheetz, 2006; Sukharev and Sachs, 2012). In turn, it has long been known that integrin/ligand binding is affected by divalent cations (also Ca^2+^) (Zhang and Chen, 2012). Local changes in calcium concentration influence integrin adhesion dynamics in growth cones and axon guidance in a calpain/talin-dependent manner (Kerstein et al., 2017), mediated e.g., by Piezo1/Fam38A (McHugh et al., 2010). However, despite this acknowledged role of calcium signals in neuronal differentiative processes, the exact spatiotemporal regulation and impact of calcium signaling is rather complex and intricate with many details still elusive (Gomez and Zheng, 2006; Leclerc et al., 2012; Kerstein et al., 2013; Toth et al., 2016).

**Figure 6:**
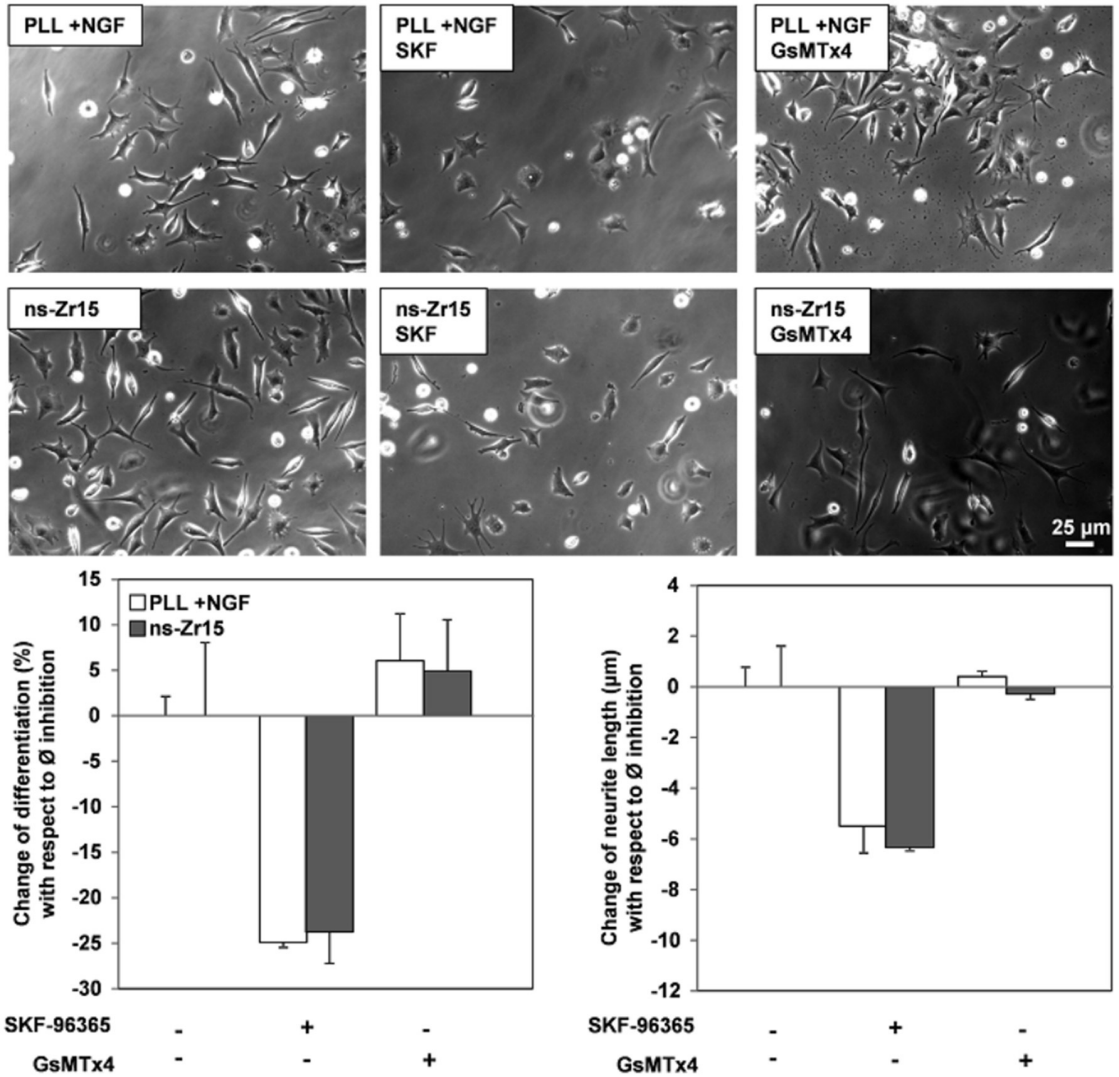
Impact of treatment with drugs affecting different types of calcium channels (SKF-96365 and GsMTx4). The phase contrast and the graph show the effect of the drugs SKF-96365 (15 μM) and GsMTx4 (10 μM), affecting transient receptor potential cation channels (TRPC), respectively stretch-activated channels (SAC), on PC12 differentiation grown in the PLL +NGF and ns-Zr15 condition. The graph represents the global statistics of two independent experiments with the change of differentiation rate (left graph) and neurite length (right graph) in comparison to the corresponding condition Ø inhibition (white bars: PLL + NGF, gray bars: ns-Zr15). The bars represent the average (mean ± s.d.) of the two experiments (comprising in total 434–650 cells and 108–387 neurites quantified).

To study the involvement of mechanosensitive channel types in our experimental context, we used the inhibitors SKF-96365 for transient receptor potential cation channels (TRPC) and GsMTx4 for stretch-activated channels (SAC, such as e.g., Piezo). With respect to the canonical NGF-stimulated outgrowth our results were in line with findings published by others, i.e., the two inhibitors showed opposing effects on neurite outgrowth (Jacques-Fricke et al., 2006; Gottlieb et al., 2010; Kerstein et al., 2013). SKF-96365 impeded differentiation and neurite outgrowth, whereas GsMTx4 had a minor differentiation-enhancing effect (Figure 6). This is consistent with the reported crosstalk between NGF/TrkA and TRPC-mediated calcium signaling (De Bernardi et al., 1996; Cohen et al., 2015). More interestingly and despite its independence of NGF/TrkA activation (Schulte et al., 2016a), the outcome was practically in the same range for the nanotopography-promoted neuritogenesis (Figure 6) suggesting a contribution of calcium signaling also in this mechanotransductively fostered differentiation.

This reflected in the phosphoproteomic analysis. Independent of whether the neuritogenesis-inducing stimulus was NGF or the nanotopography, numerous proteins with reported roles in the regulation of calcium signaling or homeostasis were differentially expressed and/or phosphorylated in the differentiated cells, compared to the PLL condition [e.g., Cacna1g (Perez-Reyes et al., 1998; Frank, 2014), collagen XVIII (Wang et al., 2014), Slc24a3 (Lytton, 2007), Camta1 (Henrich et al., 2011), Ubr4 (Nakatani et al., 2005; Belzil et al., 2013; Parsons et al., 2015), eIF3e (Green et al., 2007); Tables S1–S6, S13–S22 and details in Table 9; Ptprf/LAR (den Hertog et al., 1994; Johnson and Van Vactor, 2003; Dunah et al., 2005; Kuo et al., 2011; Um and Ko, 2013; Sarhan et al., 2016), G3bp1 (Martin et al., 2013); details in Table 7]. However, the phosphoproteomic data insinuate potential differences in calcium signaling-involved proteins between the ns-Zr15 and the NGF condition. Some proteins e.g., are only upregulated in ns-Zr15_vs_PLL (Table S3) and not in NGF_vs_PLL (Table S1), such as annexin A8, thrombospondin-1, and versican. Annexin A8 is particularly interesting regarding mechanotransduction due to the fact that this protein is recruited in a Ca^2+^-dependent manner to PIP_2_-rich membrane domains at F-actin accumulation sites. It might therefore be important in the organization of specific membrane/cytoskeleton contacts (Goebeler et al., 2006). To our best knowledge, roles of annexin A8 in neuronal cells have not been reported yet. The ECM glycoproteins thrombospondin-1 and versican; beyond the already mentioned involvement in IAC dynamics and neurito/synaptogenesis (Table 2), bind calcium which affects their structure/function and participates in the regulation of calcium concentration (Ney et al., 2006; Carlson et al., 2008).

**Table 9:** Comparison among all conditions with focus on proteins involved in calcium signaling and homeostasis.

**Associated to** **Calcium signaling/homeostasis-related proteins affected by the cell/nanotopography interaction**			
**Protein name**	**Reported protein functions**	**References**	**Category**
Cacna1g (voltage-dependent, T-type, α 1G subunit calcium channel)	Cacna1g is a member of the CaV family, a protein family which is essential for the postsynaptic homeostasis of synaptic plasticity and thus the proper functioning of synapses.	Perez-Reyes et al., 1998; Frank, 2014	Channel
Collagen XVIII	The drosophila homologue of collagen XVIII/endostatin have been found to be involved in the homeostatic presynaptic plasticity by interacting with CaV2.1 calcium channels and regulating calcium influx.	Wang et al., 2014	Extracellular matrix
Slc24a3 (solute carrier family 24, sodium/potassium/calcium exchanger, member 3)/NCKX3 (Na(+)/K(+)/Ca(2+)-exchange protein 3)	The potassium-dependent sodium/calcium exchanger slc24a3/NCKX3 exchanges, as the name implies, in a potassium-dependent manner sodium or calcium primarily in neurons.	Lytton, 2007	Channel
CAMTA1 (Calmodulin-binding transcription activator 1)	CAMTA1, which is deleted in neuroblastoma, induces neurites and expression of neuronal differentiation markers.	Henrich et al., 2011	Transcription factor
Ubr4 (E3 ubiquitin-protein ligase)/p600	Ubr4 is involved in membrane morphogenesis and integrin signaling. It is required for neuronal survival in a calcium/calmodulin-dependent mechanism. The protein has versatile functions in the CNS, calcium signaling and cytoskeletal organization. The phosphorylation level of p600 at cyclin-dependent consensus site varies during cell cycle. The function of this phosphorylation, in particular in a neuronal context, is unknown, though.	Nakatani et al., 2005; Belzil et al., 2013; Parsons et al., 2015	Neuronal survival, membrane remodeling, integrin signaling, cytoskeletal organization
eIF3e (eukaryotic initiation factor 3 subunit)	This tumor suppressor has been shown to contribute to the trafficking of CaV calcium channels and therefore the regulation regulation of calcium influx and intracellular calcium levels by these type of channels.	Green et al., 2007	Transcription, trafficking
S100A10 (S100 calcium-binding protein A10)	This protein is, as the only member of the S100 family, not capable of Ca^2+^ binding. It is nevertheless known to be implicated in calcium signaling, e.g., by regulating the trafficking of ion channels (also TRP). It participates also to the transport of neurotransmitter (receptors). Furthermore, together with AHNAK, it is involved in governing f-actin and cell membrane organization.	Benaud et al., 2004; Rescher and Gerke, 2008; Jung et al., 2010	Trafficking, membrane remodeling, cytoskeletal organization

*Selected proteins differentially expressed after cell/nanotopography interaction that have reported functions in calcium signaling/homeostasis*.

Other proteins related to calcium signaling can be found upregulated comparing directly ns-Zr15_vs_NGF (Table S2), such as Ahnak, Fat4 (protocadherin 4) and Prkcsh/PKC substrate 80K-H. Ahnak is a scaffolding protein that partakes in versatile cellular processes, many in fact related to calcium signaling (Davis et al., 2014) and/or membrane morphogenesis (together with S100A10) (Benaud et al., 2004; Lorusso et al., 2006). In the neuronal context, it is also a marker of enlargeosomes. These exocytic vesicles can contribute to the membrane supply for neurite outgrowth in a REST-regulated manner (Borgonovo et al., 2002; Racchetti et al., 2010; Schulte et al., 2010). Fat/Protocadherin 4 belongs to the calcium-dependent cadherin cell adhesion protein family and has been reported to be involved in the regulation of neuroprogenitor proliferation and differentiation upstream of YAP. Fat4 downregulation lowers differentiation of neuroprogenitors into neurons in the cerebellum (Cappello et al., 2013). Prkcsh/PKC substrate 80K-H, which colocalises with IP_3_R1, modulates IP_3_-induced calcium release and might therefore have a role in synaptic plasticity (Kawaai et al., 2009).

In comparison to ns-Zr25, in addition to already mentioned ones (here or in the tables, such as e.g., Fat4, Cacna1g, G3BP1), the S100 calcium-binding protein A10 (Benaud et al., 2004; Rescher and Gerke, 2008; Jung et al., 2010) differentially phosphorylated in the cells on ns-Zr15 (details in Table 9).

In summary, Ca^2+^ signaling is important for both, NGF- and nanotopography-triggered neuritogenesis, but the proteomic data suggest that the cell/nanotopography interaction might influence some specific proteins prominently involved in calcium homeostasis and/or signaling.

## Conclusions

In recent years the relevance of microenvironmental and cellular mechanobiological aspects with respect to differentiation processes have become evident (Geiger et al., 2009; Wang et al., 2009; Dalby et al., 2014; Jansen et al., 2015), particularly in the neuronal context (Franze et al., 2013). However, many details regarding the mechanosensing of the microenvironment, the signal transmission, and integration within the mechanotransductive sequence remain elusive (Dalby et al., 2014; Humphries et al., 2015; Jansen et al., 2015). In previous publications we have introduced how ECM-like nanotopographical features of cluster-assembled titania and zirconia sufaces, produced by SCBD, can foster important events in neuronal differentiation (such as e.g., neuritogenesis; Schulte et al., 2016a,b, synaptogenesis and network maturation; Schulte et al., 2016b) through mechanotransductive pathways.

The presently adopted quantitative proteomic approach (associated to a systematic characterization) challenges PC12 cells with diverse experimental situations that address the impact of substrate nanotopography and/or cellular biomechanics on neuronal cell fate. The analyzed conditions comprised the canonical PC12 cell differentiation setting with a biochemical stimulus (PLL ±NGF; i.e., PLL and NGF), three different zirconia surface topographies with distinct nanoscale roughness parameters (flat-Zr, ns-Zr15, ns-Zr25) and treatments that affect the tensional state of the cell (ns-Zr15 hypo, PLL hyper). This approach enabled us to acquire an extensive molecular image of the processes and pathways that are sensitive to changes in the microenvironmental nanotopography and/or cellular nanomechanical properties, and to identify potential key elements therein. The data thus provide various starting points and indications on how the nanotopographical sensitivity is achieved and integrated into signaling pathways.

We found various common, but also distinctive features in the protein expression and phosphorylation profiles comparing the canonical biochemically (NGF-)induced neuronal differentiation and the one triggered by the cell/nanotopography interaction. In this neuron-like PC12 cell model the mechanotransductive stimulus provided by an appropriate nanotopography is alone sufficient to achieve the necessary change in the cellular program that implements the neuronal differentiation. There are indications in the phosphoproteomic data that the nanotopographical stimulus is even more effective. A robust engagement of proteins involved in cell morphology, cellular assembly and organization, and cellular movement (see IPA, Table 3) was observed and many proteins with acknowledged roles related to IAC/mechanotransduction were altered. In addition, versatile proteins with tasks in neuronal functioning and differentiation have been identified to be modulated in the nanotopography setting; as well as several proteins essentially involved in epigenetic and (post-)transcriptional regulation during neuronal differentiation (Tables 1, 2, 5, 7). This is in line with the hypothesis of a potential epigenetic regulation of cell reprogramming and differentiation dependent on cellular mechanics and microenvironmental cues (such as in this case surface nanotopography) (Downing et al., 2013; Crowder et al., 2016).

We have seen on the morphological level that only specific roughness parameters of the cluster-assembled zirconia surfaces (for PC12 cells ns-Zr15) provide appropriate biophysical cues to gain full neuritogenesis. An increased nanotopography roughness (ns-Zr25) instead leads to an only partial effect on neuritogenesis (Schulte et al., 2016a). In line with this, the proteomic analysis demonstrated that there is a partial overlap in the molecular alterations between ns-Zr15 and ns-Zr25, compared to NGF. Yet, the comparison revealed and specified also some decisive differences regarding proteins important for integrin activation and cytoskeletal organization. Notably, the IPA emphasized the importance of IAC in the mechanosensing to interpret the distinct natures of the topographies as ILK (integrin-linked kinase) signaling is the only decreased pathway (Table 6). This is striking because ILK signaling is essentially involved in determining the molecular architecture of IAC and its signaling is sensitive to variations in ligand spacing/density. The proteomic data suggests furthermore that the increase in roughness starts to cause cellular stress in this PC12 cell model.

In summary, many of the proteins found to be altered at the expression and/or phosphorylation level (Tables 2, 5, 7) can be associated with the following categories which all have high relevance with respect to mechanotransductive signaling: cell/cell adhesion, glycocalyx and ECM, integrin activation and membrane/f-actin linkage, cell/substrate interaction and IAC, actomyosin organization/cellular mechanics, and nuclear organization and transcriptional regulation. By integrating the dissected alterations into a potential context, a complex nanotopography-sensitive network with broad crosstalk opportunities crystallizes, capable of regulating the cell/microenvironment interface and consequentially cellular cytoskeletal mechanics and signaling, here in control of neuronal differentiation processes (Figure 2).

This comprehensive proteomic analysis insinuates that other pathways with strong correlation to mechanotransduction, such as Wnt (Table 8), mTOR (Figures 4, 5) and Ca^2+^ signaling (Table 9, Figure 6), might also be affected by, and involved in, the nanotopography-triggered cellular processes. However, more profound future studies are required regarding these pathways.

Altogether, this proteomic-based analysis defined nanotopography-sensitive signaling hubs and key elements potentially important in the promotion of neuronal differentiation by nanotopographical cues. It delivered several interesting starting points to evaluate in more specific studies, and in a wider context with respect to the role of certain proteins in mechanotransductive signaling that regulates neuron development and maturation. In the framework of biomaterials that are based on nanoscale surface features, an in-depth understanding of the impact of cell/nanotopography interaction on cellular processes and fate is the indispensable prerequisite. An improved insight might help to harness and effectively control the potential of these biomaterials in biomedical applications. Vice versa, information obtained by advanced biomaterial approaches could provide conclusions for a better comprehension of the difficult to access *in vivo* mode of operation of microenvironmental and cellular mechanobiological processes, e.g., regarding epigenetic regulation.

## Author contributions

The project was mostly conceived by CS and GT. EM, CS, and GT wrote the principal part of the manuscript and realized the figures. The proteomic approach and related data analyses were done by EM, SN, FG, AN, CS, and GT. CS performed the cell biological inhibition experiments and corresponding quantifications. CP executed the fabrication of the nanostructured surfaces by SCBD and flat-Zr by electron beam evaporation. CL and PM participated in the project conception/creation and the realization of the manuscript, contributing moreover reagents, materials, analysis tools and considerations regarding the nanotechnological aspects of the work.

### Conflict of interest statement

The authors declare that the research was conducted in the absence of any commercial or financial relationships that could be construed as a potential conflict of interest.

## Supplementary material

Figure S1 Induction of neurite outgrowth in PC12 cells by hyperosmotic shock on PLL. The phase contrast images in the panel illustrate the response of PC12 cells on PLL after applying a hyperosmotic shock (15 min, 150 mM sucrose), with the conditions PLL ± NGF as references. The graph represents the global statistic of two independent experiments (in total 343–398 cells and 226–251 neurites quantified). The bars show the average (mean) change compared to the PLL –NGF condition (white bars: differentiation rate; black bars: neurite length) and are flanked by the standard deviation (s.d.).

Figure S2 Comparison of the neurite morphology of PC12 cells in the between NGF and ns-Zr15 condition. The graph shows on the left the average number neurites per differentiated PC12 cell grown on PLL and stimulated with NGF (white bar) or interacting with ns-Zr15 (gray bar). The bars are flanked by the standard deviation (s.d.). On the right, the bars demonstrate the frequency of the different categories indicating the number of neurites per differentiated cells in the two conditions. Representative examples of the cell morphology in these conditions can be found in the phase contrast images of Figures 1, 5, 6. In total 160 cells from 8 independent experiments were quantified.

Figure S3 Comparison of PLL, NGF and ns-Zr15. **(A)** Venn diagram; **(B)** work flow; **(C)** Vulcano plots; **(D)** ClueGo analysis of proteins upregulated or expressed only in cells grown on ns-Zr15_vs_NGF. Functional grouping was based on *p* ≤ 0.05 and at least two counts.

Figure S4 Comparison of NGF, ns-Zr15, ns-Zr25. **(A)** Venn diagram; **(B)** work flow

Table S1 Proteins differently expressed comparing NGF_vs_PLL. List of the proteins annotated with Perseus software. Categorical annotation was supplied in form of Gene Ontology (GO) biological process (BP), molecular function (MF), cellular component (CC), keywords as well as in participation in a KEGG pathway, Reactome and molecular complexes (CORUM).

Table S2 Proteins differently expressed comparing NGF_vs_ns-Zr15. List of the proteins annotated with Perseus software. Categorical annotation was supplied in form of Gene Ontology (GO) biological process (BP), molecular function (MF), cellular component (CC), keywords as well as in participation in a KEGG pathway, Reactome and molecular complexes (CORUM). Proteins that are of particular interest regarding a potential connection of IAC/mechanotransductive signaling and neuronal differentiation processes in the nanotopography-induced setting are marked in gray.

Table S3 Proteins differently expressed comparing ns-Zr15_vs_PLL. List of the proteins annotated with Perseus software. Categorical annotation was supplied in form of Gene Ontology (GO) biological process (BP), molecular function (MF), cellular component (CC), keywords as well as in participation in a KEGG pathway, Reactome and molecular complexes (CORUM). **X** = proteins differentially expressed in the same manner in ns-Zr15_vs_flat-Zr and ns-Zr15_vs_PLL. In gray, proteins differentially expressed in the same manner in NGF_vs_PLL and ns-Zr15_vs_PLL.

Table S4 Proteins expressed only in NGF (with respect to the comparison PLL, NGF, ns-Zr15). List of the proteins annotated with Perseus software. Categorical annotation was supplied in form of Gene Ontology (GO) biological process (BP), molecular function (MF), cellular component (CC), keywords as well as in participation in a KEGG pathway and molecular complexes (CORUM).

Table S5 Proteins expressed only in ns-Zr15. List of the proteins annotated with Perseus software. Categorical annotation was supplied in form of Gene Ontology (GO) biological process (BP), molecular function (MF), cellular component (CC), keywords as well as in participation in a KEGG pathway and molecular complexes (CORUM). Proteins that are of particular interest regarding a potential connection of IAC/mechanotransductive signaling and neuronal differentiation processes in the nanotopography-induced setting are marked in gray.

Table S6 Proteins expressed only in PLL. List of the proteins annotated with Perseus software. Categorical annotation was supplied in form of Gene Ontology (GO) biological process (BP), molecular function (MF), cellular component (CC), keywords as well as in participation in a KEGG pathway and molecular complexes (CORUM).

Table S7 Proteins differently expressed comparing ns-Zr25_vs_ns-Zr15. List of the proteins annotated with Perseus software. Categorical annotation was supplied in form of Gene Ontology (GO) biological process (BP), molecular function (MF), cellular component (CC), keywords as well as in participation in a KEGG pathway and molecular complexes (CORUM).

Table S8 Proteins differently expressed comparing ns-Zr25_vs_NGF. List of the proteins annotated with Perseus software. Categorical annotation was supplied in form of Gene Ontology (GO) biological process (BP), molecular function (MF), cellular component (CC), keywords as well as in participation in a KEGG pathway, Reactome and molecular complexes (CORUM). Proteins found to be upregulated in ns-Zr25_vs_NGF and also up-regulated in the comparison ns-Zr15_vs_NGF or ns-Zr15_vs_PLL are marked in gray.

Table S9 Proteins differently expressed comparing ns-Zr15_vs_NGF. List of the proteins annotated with Perseus software. Categorical annotation was supplied in form of Gene Ontology (GO) biological process (BP), molecular function (MF), cellular component (CC), keywords as well as in participation in a KEGG pathway and molecular complexes (CORUM).

Table S10 Proteins expressed only in NGF (with respect to the comparison NGF, ns-Zr15, ns-Zr25). List of the proteins annotated with Perseus software. Categorical annotation was supplied in form of Gene Ontology (GO) biological process (BP), molecular function (MF), cellular component (CC), keywords as well as in participation in a KEGG pathway and molecular complexes (CORUM).

Table S11 Proteins expressed only in ns-Zr15 (with respect to the comparison NGF, ns-Zr15, ns-Zr25). List of the proteins annotated with Perseus software. Categorical annotation was supplied in form of Gene Ontology (GO) biological process (BP), molecular function (MF), cellular component (CC), keywords as well as in participation in a KEGG pathway and molecular complexes (CORUM).

Table S12 Proteins expressed only in ns-Zr25. List of the proteins annotated with Perseus software. Categorical annotation was supplied in form of Gene Ontology (GO) biological process (BP), molecular function (MF), cellular component (CC), keywords as well as in participation in a KEGG pathway and molecular complexes (CORUM).

Table S13 Proteins differently phosphorylated in NGF_vs_PLL. The table lists all phosphorylated proteins annotated with Perseus software. Annotated terms include GOBP, GOMF, GOCC, Keywords, KEGG, CORUM, together with the enriched sequence motifs of phosphopeptides data set.

Table S14 Proteins differently phosphorylated in ns-Zr15_vs_PLL. The table lists all phosphorylated proteins annotated with Perseus software. Annotated terms include GOBP, GOMF, GOCC, Keywords, KEGG, CORUM, together with the enriched sequence motifs of phosphopeptides data set.

Table S15 Proteins phosphorylated only in NGF. The table lists all phosphorylated proteins annotated with Perseus software. Annotated terms include GOBP, GOMF, GOCC, Keywords, KEGG, CORUM, together with the enriched sequence motifs of phosphopeptides data set.

Table S16 Proteins phosphorylated only in ns-Zr15. The table lists all phosphorylated proteins annotated with Perseus software. Annotated terms include GOBP, GOMF, GOCC, Keywords, KEGG, CORUM, together with the enriched sequence motifs of phosphopeptides data set.

Table S17 Proteins differently phosphorylated in ns-Zr15_vs_flat-Zr. The table lists all phosphorylated proteins annotated with Perseus software. Annotated terms include GOBP, GOMF, GOCC, Keywords, KEGG, CORUM, together with the enriched sequence motifs of phosphopeptides data set.

Table S18 Proteins exclusively phosphorylated in ns-Zr15 (with respect to the comparison PLL, NGF, ns-Zr15, flat-Zr). The table lists all phosphorylated proteins annotated with Perseus software. Annotated terms include GOBP, GOMF, GOCC, Keywords, KEGG, CORUM, together with the enriched sequence motifs of phosphopeptides data set.

Table S19 Proteins exclusively phosphorylated in flat-Zr. The table lists all phosphorylated proteins annotated with Perseus software. Annotated terms include GOBP, GOMF, GOCC, Keywords, KEGG, CORUM, together with the enriched sequence motifs of phosphopeptides data set.

Table S20 Proteins differently phosphorylated in ns-Zr15_vs_ns-Zr25. The table lists all phosphorylated proteins annotated with Perseus software. Annotated terms include GOBP, GOMF, GOCC, Keywords, KEGG, CORUM, together with the enriched sequence motifs of phosphopeptides data set.

Table S21 Proteins differently phosphorylated in ns-Zr25_vs_NGF. The table lists all phosphorylated proteins annotated with Perseus software. Annotated terms include GOBP, GOMF, GOCC, Keywords, KEGG, CORUM, together with the enriched sequence motifs of phosphopeptides data set.

Table S22 Proteins exclusively phosphorylated in ns-Zr15 (with respect to the comparison ns-Zr15, ns-Zr25). The table lists all phosphorylated proteins annotated with Perseus software. Annotated terms include GOBP, GOMF, GOCC, Keywords, KEGG, CORUM, together with the enriched sequence motifs of phosphopeptides data set.

Table S23 Proteins differently expressed comparing ns-Zr15 hypo_vs_ns-Zr15. List of the proteins annotated with Perseus software. Categorical annotation was supplied in form of Gene Ontology (GO) biological process (BP), molecular function (MF), cellular component (CC), keywords as well as in participation in a KEGG pathway and molecular complexes (CORUM). **X** = proteins with an opposed alteration of the expression level comparing ns-Zr15_vs_flat-Zr with ns-Zr15 hypo_vs_ns-Zr15; **Y** = proteins altered in the same way in ns-Zr15_vs_flat-Zr and ns-Zr15 hypo_vs_ns-Zr15.

Table S24 Proteins expressed only in ns-Zr15 (with respect to the comparison ns-Zr15, ns-Zr15 hypo). List of the proteins annotated with Perseus software. Categorical annotation was supplied in form of Gene Ontology (GO) biological process (BP), molecular function (MF), cellular component (CC), keywords as well as in participation in a KEGG pathway and molecular complexes (CORUM).

Table S25 Proteins differently expressed comparing PLL hyper_vs_PLL. List of the proteins annotated with Perseus software. Categorical annotation was supplied in form of Gene Ontology (GO) biological process (BP), molecular function (MF), cellular component (CC), keywords as well as in participation in a KEGG pathway and molecular complexes (CORUM). **x** = proteins with a similar alteration of the expression level comparing ns-Zr15_vs_flat-Zr and PLL hyper_vs_PLL; **X** = proteins altered in an opposite manner in ns-Zr15_vs_flat-Zr and PLL hyper_vs_PLL.

## Abbreviations

AFM: Atomic force microscopy
ECM: Extracellular matrix
FA: Focal adhesion(s)
FBS: Fetal bovine serum
FC: Focal complex(es)
HS: Horse serum
IAC: Integrin adhesion complex(es)
IPA: Ingenuity pathway analysis
min: Minutes
mM: Milli molar
NGF: Nerve growth factor
ns-Zr: Nanostructured zirconia
PCA: Principal component analysis
PLL: Poly-L-lysine
RMS: Root mean square
SAC: Stretch-activated channels
SCBD: Supersonic cluster beam deposition
SD: Standard deviation
TrkA: Tropomyosin receptor kinase A
TRPC: Transient receptor potential cation channels.

## References

[B1] Abraham A. B. Bronstein R. Reddy A. S. Maletic-Savatic M. Aguirre A. Tsirka S. E. (2013). Aberrant neural stem cell proliferation and increased adult neurogenesis in mice lacking chromatin protein HMGB2. PloS ONE 8:e84838. 10.1371/journal.pone.0084838 24391977 PMC3877347

[B2] Adams J. C. Kureishy N. Taylor A. L. (2001). A role for syndecan-1 in coupling fascin spike formation by thrombospondin-1. J. Cell Biol. 152, 1169–1182. 10.1083/jcb.152.6.1169 11257118 PMC2199199

[B3] Alahari S. K. Lee J. W. Juliano R. L. (2000). Nischarin, a novel protein that interacts with the integrin alpha5 subunit and inhibits cell migration. J. Cell Biol. 151, 1141–1154. 10.1083/jcb.151.6.1141 11121431 PMC2190593

[B4] Alahari S. K. Reddig P. J. Juliano R. L. (2004). The integrin-binding protein Nischarin regulates cell migration by inhibiting PAK. EMBO J. 23, 2777–2788. 10.1038/sj.emboj.7600291 15229651 PMC514951

[B5] Anitei M. Hoflack B. (2012). Bridging membrane and cytoskeleton dynamics in the secretory and endocytic pathways. Nat. Cell Biol. 14, 11–19. 10.1038/ncb2409 22193159

[B6] Asperti C. Astro V. Totaro A. Paris S. de Curtis I. (2009). Liprin-alpha1 promotes cell spreading on the extracellular matrix by affecting the distribution of activated integrins. J. Cell Sci. 122(Pt 18), 3225–3232. 10.1242/jcs.054155 19690048

[B7] Asperti C. Pettinato E. de Curtis I. (2010). Liprin-alpha1 affects the distribution of low-affinity beta1 integrins and stabilizes their permanence at the cell surface. Exp. Cell Res. 316, 915–926. 10.1016/j.yexcr.2010.01.017 20096687

[B8] Bae B. I. Tietjen I. Atabay K. D. Evrony G. D. Johnson M. B. Asare E.. (2014). Evolutionarily dynamic alternative splicing of GPR56 regulates regional cerebral cortical patterning. Science 343, 764–768. 10.1126/science.1244392 24531968 PMC4480613

[B9] Bargsted L. Hetz C. Matus S. (2016). ERp57 in neurodegeneration and regeneration. Neural Regen. Res. 11, 232–233. 10.4103/1673-5374.177722 27073369 PMC4810980

[B10] Barker T. H. Pallero M. A. MacEwen M. W. Tilden S. G. Woods A. Murphy-Ullrich J. E.. (2004). Thrombospondin-1-induced focal adhesion disassembly in fibroblasts requires Thy-1 surface expression, lipid raft integrity, and Src activation. J. Biol. Chem. 279, 23510–23516. 10.1074/jbc.M402169200 15033989

[B11] Belzil C. Neumayer G. Vassilev A. P. Yap K. L. Konishi H. Rivest S.. (2013). A Ca2+-dependent mechanism of neuronal survival mediated by the microtubule-associated protein p600. J. Biol. Chem. 288, 24452–24464. 10.1074/jbc.M113.483107 23861403 PMC3750145

[B12] Benaud C. Gentil B. J. Assard N. Court M. Garin J. Delphin C.. (2004). AHNAK interaction with the annexin 2/S100A10 complex regulates cell membrane cytoarchitecture. J. Cell Biol. 164, 133–144. 10.1083/jcb.200307098 14699089 PMC2171952

[B13] Blanco S. Dietmann S. Flores J. V. Hussain S. Kutter C. Humphreys P.. (2014). Aberrant methylation of tRNAs links cellular stress to neuro-developmental disorders. EMBO J. 33, 2020–2039. 10.15252/embj.201489282 25063673 PMC4195770

[B14] Borghi F. Sogne E. Lenardi C. Podestà A. Merlini M. Ducati C. (2016). Cluster-assembled cubic zirconia films with tunable and stable nanoscale morphology against thermal annealing. J. Appl. Phys. 120:055302 10.1063/1.4960441

[B15] Borgonovo B. Cocucci E. Racchetti G. Podini P. Bachi A. Meldolesi J. (2002). Regulated exocytosis: a novel, widely expressed system. Nat. Cell Biol. 4, 955–962. 10.1038/ncb888 12447386

[B16] Boyne L. J. Fischer I. Shea T. B. (1996). Role of vimentin in early stages of neuritogenesis in cultured hippocampal neurons. Int. J. Dev. Neurosci. 14, 739–748. 10.1016/S0736-5748(96)00053-6 8960981

[B17] Bradley R. S. Cowin P. Brown A. M. (1993). Expression of Wnt-1 in PC12 cells results in modulation of plakoglobin and E-cadherin and increased cellular adhesion. J. Cell Biol. 123(6 Pt 2), 1857–65. 10.1083/jcb.123.6.1857 8276903 PMC2290857

[B18] Buchman J. J. Durak O. Tsai L. H. (2011). ASPM regulates Wnt signaling pathway activity in the developing brain. Genes Dev. 25, 1909–1914. 10.1101/gad.16830211 21937711 PMC3185963

[B19] Cang Y. Zhang J. Nicholas S. A. Bastien J. Li B. Zhou P.. (2006). Deletion of DDB1 in mouse brain and lens leads to p53-dependent elimination of proliferating cells. Cell 127, 929–940. 10.1016/j.cell.2006.09.045 17129780

[B20] Cao J. P. Yu J. K. Li C. Sun Y. Yuan H. H. Wang H. J.. (2008). Integrin beta1 is involved in the signaling of glial cell line-derived neurotrophic factor. J. Comp. Neurol. 509, 203–210. 10.1002/cne.21739 18465789

[B21] Cappello S. Gray M. J. Badouel C. Lange S. Einsiedler M. Srour M.. (2013). Mutations in genes encoding the cadherin receptor-ligand pair DCHS1 and FAT4 disrupt cerebral cortical development. Nat. Genet. 45, 1300–1308. 10.1038/ng.2765 24056717

[B22] Carlson C. B. Lawler J. Mosher D. F. (2008). Structures of thrombospondins. Cell Mol. Life Sci. 65, 672–686. 10.1007/s00018-007-7484-1 18193164 PMC2578829

[B23] Castillo V. Oñate M. Woehlbier U. Rozas P. Andreu C. Medinas D. (2015). Functional role of the Disulfide Isomerase ERp57 in Axonal Regeneration. PLOS ONE. 10:e0136620 10.1371/journal.pone.0136620 26361352 PMC4567344

[B24] Chen W. Shao Y. Li X. Zhao G. Fu J. (2014). Nanotopographical surfaces for stem cell fate control: engineering mechanobiology from the bottom. Nano Today 9, 759–784. 10.1016/j.nantod.2014.12.002 25883674 PMC4394389

[B25] Chen W. Villa-Diaz L. G. Sun Y. Weng S. Kim J. K. Lam R. H. W.. (2012). Nanotopography influences adhesion, spreading, and self-renewal of human embryonic stem cells. ACS Nano 6, 4094–4103. 10.1021/nn3004923 22486594 PMC3358529

[B26] Cohen M. R. Johnson W. M. Pilat J. M. Kiselar J. DeFrancesco-Lisowitz A. Zigmond R. E.. (2015). Nerve growth factor regulates transient receptor potential vanilloid 2 via extracellular signal-regulated kinase signaling to enhance neurite outgrowth in developing neurons. Mol. Cell Biol. 35, 4238–4252. 10.1128/MCB.00549-15 26416880 PMC4648816

[B27] Cosker K. E. Segal R. A. (2014). Neuronal Signaling through Endocytosis. Cold Spring Harb Perspect Biol. 6:a020669. 10.1101/cshperspect.a020669 24492712 PMC3941234

[B28] Cranford S. W. de Boer J. van Blitterswijk C. Buehler M. J. (2013). Materiomics: an -omics approach to biomaterials research. Adv. Mater. Deerfield Beach Fla. 25, 802–824. 10.1002/adma.201202553 23297023

[B29] Crowder S. W. Leonardo V. Whittaker T. Papathanasiou P. Stevens M. M. (2016). Material cues as potent regulators of epigenetics and stem cell function. Cell Stem Cell 18, 39–52. 10.1016/j.stem.2015.12.012 26748755 PMC5409508

[B30] Dalby M. J. Gadegaard N. Oreffo R. O. C. (2014). Harnessing nanotopography and integrin-matrix interactions to influence stem cell fate. Nat. Mater. 13, 558–569. 10.1038/nmat3980 24845995

[B31] Dasgupta S. Lonard D. M. O'Malley B. W. (2014). Nuclear receptor coactivators: master regulators of human health and disease. Annu. Rev. Med. 65, 279–292. 10.1146/annurev-med-051812-145316 24111892 PMC4327818

[B32] da Silva J. S. Dotti C. G. (2002). Breaking the neuronal sphere: regulation of the actin cytoskeleton in neuritogenesis. Nat. Rev. Neurosci. 3, 694–704. 10.1038/nrn918 12209118

[B33] Davis T. A. Loos B. Engelbrecht A.-M. (2014). AHNAK: the giant jack of all trades. Cell Signal. 26, 2683–2693. 10.1016/j.cellsig.2014.08.017 25172424

[B34] De Bernardi M. A. Rabins S. J. Colangelo A. M. Brooker G. Mocchetti I. (1996). TrkA mediates the nerve growth factor-induced intracellular calcium accumulation. J. Biol. Chem. 271, 6092–6098. 10.1074/jbc.271.11.6092 8626395

[B35] Deng T. Zhu Z. I. Zhang S. Leng F. Cherukuri S. Hansen L.. (2013). HMGN1 modulates Nucleosome occupancy and DNase I Hypersensitivity at the CpG island promoters of Embryonic stem cells. Mol. Cell Biol. 33, 3377–3389. 10.1128/MCB.00435-13 23775126 PMC3753902

[B36] den Hertog J. Tracy S. Hunter T. (1994). Phosphorylation of receptor protein-tyrosine phosphatase alpha on Tyr789, a binding site for the SH3-SH2-SH3 adaptor protein GRB-2 *in vivo*. EMBO J. 13, 3020–3032. 7518772 10.1002/j.1460-2075.1994.tb06601.x PMC395191

[B37] Dhar S. S. Lee S. H. Kan P. Y. Voigt P. Ma L. Shi X.. (2012). Trans-tail regulation of MLL4-catalyzed H3K4 methylation by H4R3 symmetric dimethylation is mediated by a tandem PHD of MLL4. Genes Dev. 26, 2749–2762. 10.1101/gad.203356.112 23249737 PMC3533079

[B38] Ding Y. Li Y. Lu L. Zhang R. Zeng L. Wang L.. (2015). Inhibition of Nischarin expression promotes Neurite outgrowth through regulation of PAK activity. PLOS ONE 10:e0144948. 10.1371/journal.pone.0144948 26670864 PMC4682924

[B39] Ding Y. Milosavljevic T. Alahari S. K. (2008). Nischarin inhibits LIM kinase to regulate cofilin phosphorylation and cell invasion. Mol. Cell Biol. 28, 3742–3756. 10.1128/MCB.01832-07 18332102 PMC2423293

[B40] Downing T. L. Soto J. Morez C. Houssin T. Fritz A. Yuan F.. (2013). Biophysical regulation of epigenetic state and cell reprogramming. Nat. Mater. 12, 1154–1162. 10.1038/nmat3777 24141451 PMC9675045

[B41] Drinjakovic J. Jung H. Campbell D. S. Strochlic L. Dwivedy A. Holt C. E. (2010). E3 ligase Nedd4 promotes axon branching by downregulating PTEN. Neuron. 65, 341–357. 10.1016/j.neuron.2010.01.017 20159448 PMC2862300

[B42] Dubash A. D. Wennerberg K. García-Mata R. Menold M. M. Arthur W. T. Burridge K. (2007). A novel role for Lsc/p115 RhoGEF and LARG in regulating RhoA activity downstream of adhesion to fibronectin. J. Cell Sci. 120(Pt 22), 3989–98. 10.1242/jcs.003806 17971419

[B43] Dunah A. W. Hueske E. Wyszynski M. Hoogenraad C. C. Jaworski J. Pak D. T.. (2005). LAR receptor protein tyrosine phosphatases in the development and maintenance of excitatory synapses. Nat. Neurosci. 8, 458–467. 10.1038/nn1416 15750591

[B44] Eaton J. M. Mullins G. R. Brindley D. N. Harris T. E. (2013). Phosphorylation of lipin 1 and charge on the phosphatidic acid head group control its phosphatidic acid phosphatase activity and membrane association. J. Biol. Chem. 288, 9933–9945. 10.1074/jbc.M112.441493 23426360 PMC3617293

[B45] Eckert M. A. Santiago-Medina M. Lwin T. M. Kim J. Courtneidge S. A. Yang J. (2017). ADAM12 induction by TWIST1 promotes tumor invasion and metastasis via regulation of invadopodia and focal adhesions. J. Cell Sci. 130, 2036-2048. 10.1242/jcs.198200 28468988 PMC5482979

[B46] Eggenschwiler J. T. Espinoza E. Anderson K. V. (2001). Rab23 is an essential negative regulator of the mouse Sonic hedgehog signalling pathway. Nature 412, 194–198. 10.1038/35084089 11449277

[B47] Elad N. Volberg T. Patla I. Hirschfeld-Warneken V. Grashoff C. Spatz J. P.. (2013). The role of integrin-linked kinase in the molecular architecture of focal adhesions. J. Cell Sci. 126(Pt 18), 4099–4107. 10.1242/jcs.120295 23843624

[B48] Essmann C. L. Martinez E. Geiger J. C. Zimmer M. Traut M. H. Stein V.. (2008). Serine phosphorylation of ephrinB2 regulates trafficking of synaptic AMPA receptors. Nat. Neurosci. 11, 1035–1043. 10.1038/nn.2171 19160501

[B49] Evans T. M. Ferguson C. Wainwright B. J. Parton R. G. Wicking C. (2003). Rab23, a negative regulator of hedgehog signaling, localizes to the plasma membrane and the endocytic pathway. Traffic 4, 869–884. 10.1046/j.1600-0854.2003.00141.x 14617350

[B50] Ezratty E. J. Bertaux C. Marcantonio E. E. Gundersen G. G. (2009). Clathrin mediates integrin endocytosis for focal adhesion disassembly in migrating cells. J. Cell Biol. 187, 733–747. 10.1083/jcb.200904054 19951918 PMC2806590

[B51] Fletcher D. A. Mullins R. D. (2010). Cell mechanics and the cytoskeleton. Nature 463, 485–492. 10.1038/nature08908 20110992 PMC2851742

[B52] Flynn K. C. (2013). The cytoskeleton and neurite initiation. Bioarchitecture 3, 86–109. 10.4161/bioa.26259 24002528 PMC4201609

[B53] Flynn K. C. Hellal F. Neukirchen D. Jacob S. Tahirovic S. Dupraz S.. (2012). ADF/cofilin-mediated actin retrograde flow directs neurite formation in the developing brain. Neuron 76, 1091–1107. 10.1016/j.neuron.2012.09.038 23259946

[B54] Frank C. A. (2014). How voltage-gated calcium channels gate forms of homeostatic synaptic plasticity. Front. Cell Neurosci. 8:40. 10.3389/fncel.2014.00040 24592212 PMC3924756

[B55] Franze K. Janmey P. A. Guck J. (2013). Mechanics in neuronal development and repair. Annu. Rev. Biomed. Eng. 15, 227–251. 10.1146/annurev-bioeng-071811-150045 23642242

[B56] Garza-Lombó C. Gonsebatt M. E. (2016). Mammalian target of rapamycin: its role in early neural development and in adult and aged brain function. Front. Cell Neurosci. 10:157. 10.3389/fncel.2016.00157 27378854 PMC4910040

[B57] Gasiorowski J. Z. Murphy C. J. Nealey P. F. (2013). Biophysical cues and cell behavior: the big impact of little things. Annu. Rev. Biomed. Eng. 15:155–176. 10.1146/annurev-bioeng-071811-150021 23862676

[B58] Gauthier N. C. Masters T. A. Sheetz M. P. (2012). Mechanical feedback between membrane tension and dynamics. Trends Cell Biol. 22, 527–535. 10.1016/j.tcb.2012.07.005 22921414

[B59] Geiger B. Spatz J. P. Bershadsky A. D. (2009). Environmental sensing through focal adhesions. Nat. Rev. Mol. Cell Biol. 10, 21–33. 10.1038/nrm2593 19197329

[B60] Geiger J. C. Lipka J. Segura I. Hoyer S. Schlager M. A. Wulf P. S.. (2014). The GRIP1/14-3-3 pathway coordinates cargo trafficking and dendrite development. Dev. Cell 28, 381–393. 10.1016/j.devcel.2014.01.018 24576423

[B61] Goebeler V. Ruhe D. Gerke V. Rescher U. (2006). Annexin A8 displays unique phospholipid and F-actin binding properties. FEBS Lett. 580, 2430–2434. 10.1016/j.febslet.2006.03.076 16638567

[B62] Goicoechea S. Orr A. W. Pallero M. A. Eggleton P. Murphy-Ullrich J. E. (2000). Thrombospondin mediates focal adhesion disassembly through interactions with cell surface calreticulin. J. Biol. Chem. 275, 36358–36368. 10.1074/jbc.M005951200 10964924

[B63] Gomez T. M. Robles E. Poo M. Spitzer N. C. (2001). Filopodial calcium transients promote substrate-dependent growth cone turning. Science 291, 1983–1987. 10.1126/science.1056490 11239161

[B64] Gomez T. M. Zheng J. Q. (2006). The molecular basis for calcium-dependent axon pathfinding. Nat. Rev. Neurosci. 7, 115–125. 10.1038/nrn1844 16429121

[B65] Gong H. Shen B. Flevaris P. Chow C. Lam S. C. T. Voyno-Yasenetskaya T. A.. (2010). G protein subunit Galpha13 binds to integrin alphaIIbbeta3 and mediates integrin ‘outside-in’ signaling. Science 327, 340–343. 10.1126/science.1174779 20075254 PMC2842917

[B66] Gottlieb P. A. Barone T. Sachs F. Plunkett R. (2010). Neurite outgrowth from PC12 cells is enhanced by an inhibitor of mechanical channels. Neurosci. Lett. 481, 115–119. 10.1016/j.neulet.2010.06.066 20600595 PMC2922024

[B67] Green E. M. Barrett C. F. Bultynck G. Shamah S. M. Dolmetsch R. E. (2007). The Tumor Suppressor eIF3e mediates calcium-dependent internalization of the L-Type calcium channel CaV1.2. Neuron. 55, 615–632. 10.1016/j.neuron.2007.07.024 17698014 PMC2384234

[B68] Groen N. Guvendiren M. Rabitz H. Welsh W. J. Kohn J. de Boer J. (2016). Stepping into the omics era: opportunities and challenges for biomaterials science and engineering. Acta Biomater. 34, 133–142. 10.1016/j.actbio.2016.02.015 26876875 PMC4830461

[B69] Gui P. Wu X. Ling S. Stotz S. C. Winkfein R. J. Wilson E.. (2006). Integrin receptor activation triggers converging regulation of Cav1.2 calcium channels by c-Src and protein kinase A pathways. J. Biol. Chem. 281, 14015–14025. 10.1074/jbc.M600433200 16554304

[B70] Gurok U. Steinhoff C. Lipkowitz B. Ropers H. H. Scharff C. Nuber U. A. (2004). Gene expression changes in the course of neural progenitor cell differentiation. J. Neurosci. 24, 5982–6002. 10.1523/JNEUROSCI.0809-04.2004 15229246 PMC6729244

[B71] Hadari Y. R. Arbel-Goren R. Levy Y. Amsterdam A. Alon R. Zakut R.. (2000). Galectin-8 binding to integrins inhibits cell adhesion and induces apoptosis. J. Cell Sci. 113(Pt 13), 2385–2397. 10852818 10.1242/jcs.113.13.2385

[B72] Han K. Yeo G. An P. Burge C. B. Grabowski P. J. (2005). A combinatorial code for splicing silencing: UAGG and GGGG motifs. PLoS Biol. 3:e158. 10.1371/journal.pbio.0030158 15828859 PMC1079783

[B73] Hart M. J. Jiang X. Kozasa T. Roscoe W. Singer W. D. Gilman A. G.. (1998). Direct stimulation of the guanine nucleotide exchange activity of p115 RhoGEF by Galpha13. Science. 280, 2112–2114. 10.1126/science.280.5372.2112 9641916

[B74] Hawkins M. Pope B. Maciver S. K. Weeds A. G. (1993). Human actin depolymerizing factor mediates a pH-sensitive destruction of actin filaments. Biochemistry 32, 9985–9993. 10.1021/bi00089a014 8399167

[B75] Heisler F. F. Lee H. K. Gromova K. V. Pechmann Y. Schurek B. Ruschkies L.. (2014). GRIP1 interlinks N-cadherin and AMPA receptors at vesicles to promote combined cargo transport into dendrites. Proc. Natl. Acad. Sci. U.S.A. 111, 5030–5035. 10.1073/pnas.1304301111 24639525 PMC3977296

[B76] Henrich K. O. Bauer T. Schulte J. Ehemann V. Deubzer H. Gogolin S.. (2011). CAMTA1, a 1p36 tumor suppressor candidate, inhibits growth and activates differentiation programs in neuroblastoma cells. Cancer Res. 71, 3142–3151. 10.1158/0008-5472.CAN-10-3014 21385898

[B77] Heuberger J. Birchmeier W. (2010). Interplay of Cadherin-Mediated cell Adhesion and Canonical Wnt signaling. Cold Spring Harb Perspect Biol. 2:a002915. 10.1101/cshperspect.a002915 20182623 PMC2828280

[B78] Heuser J. E. Reese T. S. (1973). Evidence for recycling of synaptic vesicle membrane during transmitter release at the frog neuromuscular junction. J. Cell Biol. 57, 315–344. 10.1083/jcb.57.2.315 4348786 PMC2108984

[B79] Hsia H. E. Kumar R. Luca R. Takeda M. Courchet J. Nakashima J.. (2014). Ubiquitin E3 ligase Nedd4-1 acts as a downstream target of PI3K/PTEN-mTORC1 signaling to promote neurite growth. Proc. Natl. Acad. Sci. U.S.A. 111, 13205–13210. 10.1073/pnas.1400737111 25157163 PMC4246980

[B80] Huang da. W. Sherman B. T. Lempicki R. A. (2009). Bioinformatics enrichment tools: paths toward the comprehensive functional analysis of large gene lists. Nucleic Acids Res. 37, 1–13. 10.1093/nar/gkn923 19033363 PMC2615629

[B81] Huang W. Zhu P. J. Zhang S. Zhou H. Stoica L. Galiano M.. (2013). mTORC2 controls actin polymerization required for consolidation of long-term memory. Nat. Neurosci. 16, 441–448. 10.1038/nn.3351 23455608 PMC3615448

[B82] Humphries J. D. Paul N. R. Humphries M. J. Morgan M. R. (2015). Emerging properties of adhesion complexes: what are they and what do they do? Trends Cell Biol. 25, 388–397. 10.1016/j.tcb.2015.02.008 25824971

[B83] Hussain S. Bashir Z. I. (2015). The epitranscriptome in modulating spatiotemporal RNA translation in neuronal post-synaptic function. Front. Cell Neurosci. 9:420. 10.3389/fncel.2015.00420 26582006 PMC4628113

[B84] Iguchi T. Sakata K. Yoshizaki K. Tago K. Mizuno N. Itoh H. (2008). Orphan G protein-coupled receptor GPR56 regulates neural progenitor cell migration via a G alpha 12/13 and Rho pathway. J. Biol. Chem. 283, 14469–14478. 10.1074/jbc.M708919200 18378689

[B85] Ito T. Taniguchi H. Fukagai K. Okamuro S. Kobayashi A. (2015). Inhibitory mechanism of FAT4 gene expression in response to actin dynamics during Src-induced carcinogenesis. PloS ONE 10:e0118336. 10.1371/journal.pone.0118336 25679223 PMC4334522

[B86] Jacques-Fricke B. T. Seow Y. Gottlieb P. A. Sachs F. Gomez T. M. (2006). Ca2+ Influx through Mechanosensitive channels inhibits neurite outgrowth in opposition to other influx pathways and release from intracellular stores. J. Neurosci. 26, 5656–5664. 10.1523/JNEUROSCI.0675-06.2006 16723522 PMC6675278

[B87] Jansen K. A. Donato D. M. Balcioglu H. E. Schmidt T. Danen E. H. J. Koenderink G. H. (2015). A guide to mechanobiology: where biology and physics meet. Biochim. Biophys. Acta Mol. Cell Res. 1853(11 Pt B), 3043–3052. 10.1016/j.bbamcr.2015.05.007 25997671

[B88] Jeong S. J. Luo R. Singer K. Giera S. Kreidberg J. Kiyozumi D.. (2013). GPR56 functions together with α3β1 integrin in regulating cerebral cortical development. PloS ONE 8:e68781. 10.1371/journal.pone.0068781 23874761 PMC3706371

[B89] Jia Y. Mu J. C. Ackerman S. L. (2012). Mutation of a U2 snRNA gene causes global disruption of alternative splicing and neurodegeneration. Cell 148, 296–308. 10.1016/j.cell.2011.11.057 22265417 PMC3488875

[B90] Jiang H. Shukla A. Wang X. Chen W. Bernstein B. E. Roeder R. G. (2011). Role for Dpy-30 in ES cell-fate specification by regulation of H3K4 methylation within bivalent domains. Cell 144, 513–525. 10.1016/j.cell.2011.01.020 21335234 PMC3572774

[B91] Johnson K. G. Van Vactor D. (2003). Receptor protein tyrosine phosphatases in nervous system development. Physiol. Rev. 83, 1–24. 10.1152/physrev.00016.2002 12506125

[B92] Joo E. Surka M. C. Trimble W. S. (2007). Mammalian SEPT2 Is required for scaffolding nonmuscle Myosin, I. I., and its Kinases. Dev. Cell 13, 677–690. 10.1016/j.devcel.2007.09.001 17981136

[B93] Jovceva E. Larsen M. R. Waterfield M. D. Baum B. Timms J. F. (2007). Dynamic cofilin phosphorylation in the control of lamellipodial actin homeostasis. J. Cell Sci. 120(Pt 11), 1888–1897. 10.1242/jcs.004366 17504806

[B94] Jung M. J. Murzik U. Wehder L. Hemmerich P. Melle C. (2010). Regulation of cellular actin architecture by S100A10. Exp. Cell Res. 316, 1234–1240. 10.1016/j.yexcr.2010.01.022 20100475

[B95] Katoh M. (2012). Function and cancer genomics of FAT family genes (review). Int. J. Oncol. 41, 1913–1918. 10.3892/ijo.2012.1669 23076869 PMC3583642

[B96] Kawaai K. Hisatsune C. Kuroda Y. Mizutani A. Tashiro T. Mikoshiba K. (2009). 80K-H interacts with inositol 1,4,5-trisphosphate (IP3) receptors and regulates IP3-induced calcium release activity. J. Biol. Chem. 284, 372–380. 10.1074/jbc.M805828200 18990696

[B97] Kawaguchi N. Sundberg C. Kveiborg M. Moghadaszadeh B. Asmar M. Dietrich N.. (2003). ADAM12 induces actin cytoskeleton and extracellular matrix reorganization during early adipocyte differentiation by regulating beta1 integrin function. J. Cell Sci. 116(Pt 19), 3893–904. 10.1242/jcs.00699 12915587

[B98] Kennedy M. J. Davison I. G. Robinson C. G. Ehlers M. D. (2010). Syntaxin-4 defines a domain for activity-dependent exocytosis in dendritic spines. Cell 141, 524–535. 10.1016/j.cell.2010.02.042 20434989 PMC2874581

[B99] Kerstein P. C. Jacques-Fricke B. T. Rengifo J. Mogen B. J. Williams J. C. Gottlieb P. A.. (2013). Mechanosensitive TRPC1 channels promote calpain proteolysis of talin to regulate spinal axon outgrowth. J. Neurosci. 33, 273–285. 10.1523/JNEUROSCI.2142-12.2013 23283340 PMC3539200

[B100] Kerstein P. C. Nichol R. H. Gomez T. M. (2015). Mechanochemical regulation of growth cone motility. Front. Cell Neurosci. 9:244. 10.3389/fncel.2015.00244 26217175 PMC4493769

[B101] Kerstein P. C. Patel K. M. Gomez T. M. (2017). Calpain-Mediated Proteolysis of Talin and FAK regulates Adhesion Dynamics necessary for Axon guidance. J. Neurosci. 37, 1568–1580. 10.1523/JNEUROSCI.2769-16.2016 28069919 PMC5299572

[B102] Knobloch M. Braun S. M. G. Zurkirchen L. von Schoultz C. Zamboni N. Araúzo-Bravo M. J.. (2013). Metabolic control of adult neural stem cell activity by Fasn-dependent lipogenesis. Nature 493, 226–230. 10.1038/nature11689 23201681 PMC3587167

[B103] Konishi Y. Yang L.-B. He P. Lindholm K. Lu B. Li R.. (2014). Deficiency of GDNF Receptor GFRα1 in Alzheimer's neurons results in neuronal death. J. Neurosci. 34, 13127–13138. 10.1523/JNEUROSCI.2582-13.2014 25253858 PMC4172805

[B104] Kosmaczewski S. G. Han S. M. Han B. Irving Meyer B. Baig H. S. Athar W.. (2015). RNA ligation in neurons by RtcB inhibits axon regeneration. Proc. Natl. Acad. Sci. U.S.A. 112, 8451–8456. 10.1073/pnas.1502948112 26100902 PMC4500288

[B105] Kozasa T. Jiang X. Hart M. J. Sternweis P. M. Singer W. D. Gilman A. G.. (1998). p115 RhoGEF, a GTPase activating protein for Galpha12 and Galpha13. Science. 280, 2109–2111. 10.1126/science.280.5372.2109 9641915

[B106] Kuhn T. B. Williams C. V. Dou P. Kater S. B. (1998). Laminin directs growth cone navigation via two temporally and functionally distinct calcium signals. J. Neurosci. 18, 184–194. 9412499 10.1523/JNEUROSCI.18-01-00184.1998 PMC6793400

[B107] Kuo J. C. Han X. Hsiao C.-T. Yates J. R. Waterman C. M. (2011). Analysis of the myosin-II-responsive focal adhesion proteome reveals a role for β-Pix in negative regulation of focal adhesion maturation. Nat. Cell Biol. 13, 383–393. 10.1038/ncb2216 21423176 PMC3279191

[B108] Laplante M. Sabatini D. M. (2009). mTOR signaling at a glance. J. Cell Sci. 122, 3589–3594. 10.1242/jcs.051011 19812304 PMC2758797

[B109] Launay S. Maubert E. Lebeurrier N. Tennstaedt A. Campioni M. Docagne F.. (2008). HtrA1-dependent proteolysis of TGF-beta controls both neuronal maturation and developmental survival. Cell Death Differ. 15, 1408–1416. 10.1038/cdd.2008.82 18551132

[B110] Leclerc C. Néant I. Moreau M. (2012). The calcium: an early signal that initiates the formation of the nervous system during embryogenesis. Front. Mol. Neurosci. 5:3. 10.3389/fnmol.2012.00064 22593733 PMC3351002

[B111] Lecuit T. Yap A. S. (2015). E-cadherin junctions as active mechanical integrators in tissue dynamics. Nat. Cell. Biol. 17, 533–539. 10.1038/ncb3136 25925582

[B112] Lee H. H. Tien S. C. Jou T. S. Chang Y. C. Jhong J. G. Chang Z. F. (2010). Src-dependent phosphorylation of ROCK participates in regulation of focal adhesion dynamics. J. Cell Sci. 123(Pt 19), 3368–3377. 10.1242/jcs.071555 20826462

[B113] Li Q. Lee J. A. Black D. L. (2007). Neuronal regulation of alternative pre-mRNA splicing. Nat. Rev. Neurosci. 8, 819–831. 10.1038/nrn2237 17895907

[B114] Linford A. Yoshimura S. Nunes Bastos R. Langemeyer L. Gerondopoulos A. Rigden D. J.. (2012). Rab14 and its exchange factor FAM116 link endocytic recycling and adherens junction stability in migrating cells. Dev. Cell 22, 952–966. 10.1016/j.devcel.2012.04.010 22595670 PMC3383995

[B115] Lorusso A. Covino C. Priori G. Bachi A. Meldolesi J. Chieregatti E. (2006). Annexin2 coating the surface of enlargeosomes is needed for their regulated exocytosis. EMBO J. 25, 5443–5456. 10.1038/sj.emboj.7601419 17082761 PMC1679766

[B116] Loudon R. P. Silver L. D. Yee H. F. Gallo G. (2006). RhoA-kinase and myosin II are required for the maintenance of growth cone polarity and guidance by nerve growth factor. J. Neurobiol. 66, 847–867. 10.1002/neu.20258 16673385 PMC1525020

[B117] Lytton J. (2007). Na+/Ca2+ exchangers: three mammalian gene families control Ca2+ transport. Biochem. J. 406, 365–382. 10.1042/BJ20070619 17716241

[B118] Maeda M. Hasegawa H. Hyodo T. Ito S. Asano E. Yuang H.. (2011). ARHGAP18, a GTPase-activating protein for RhoA, controls cell shape, spreading, and motility. Mol. Biol. Cell 22, 3840–3852. 10.1091/mbc.E11-04-0364 21865595 PMC3192863

[B119] Mantamadiotis T. Lemberger T. Bleckmann S. C. Kern H. Kretz O. Villalba A. M.. (2002). Disruption of CREB function in brain leads to neurodegeneration. Nat. Genet. 31, 47–54. 10.1038/ng882 11967539

[B120] Marks C. Belluscio L. Ibáñez C. F. (2012). Critical role of GFRα1 in the development and function of the main olfactory system. J. Neurosci. 32, 17306–17320. 10.1523/JNEUROSCI.1522-12.2012 23197722 PMC3655334

[B121] Martin S. Zekri L. Metz A. Maurice T. Chebli K. Vignes M.. (2013). Deficiency of G3BP1, the stress granules assembly factor, results in abnormal synaptic plasticity and calcium homeostasis in neurons. J. Neurochem. 125, 175–184. 10.1111/jnc.12189 23373770

[B122] McHugh B. J. Buttery R. Lad Y. Banks S. Haslett C. Sethi T. (2010). Integrin activation by Fam38A uses a novel mechanism of R-Ras targeting to the endoplasmic reticulum. J. Cell Sci. 123(Pt 1), 51–61. 10.1242/jcs.056424 20016066 PMC2794710

[B123] Meng X. Krishnan J. She Y. Ens W. Standing K. Wilkins J. A. (2004). Association of rasGAPSH3 binding protein 1, G3BP1, and rasGap120 with integrin containing complexes induced by an adhesion blocking antibody. J. Proteome Res. 3, 506–516. 10.1021/pr0340983 15253432

[B124] Mi H. Huang X. Muruganujan A. Tang H. Mills C. Kang D.. (2017). PANTHER version 11: expanded annotation data from Gene Ontology and Reactome pathways, and data analysis tool enhancements. Nucleic Acids Res. 45, D183–D189. 10.1093/nar/gkw1138 27899595 PMC5210595

[B125] Millán-Zambrano G. Chávez S. (2014). Nuclear functions of prefoldin. Open Biol. 4:140085. 10.1098/rsob.140085 25008233 PMC4118604

[B126] Miyake A. Mekata Y. Fujibayashi H. Nakanishi K. Konishi M. Itoh N. (2017). Brorin is required for neurogenesis, gliogenesis, and commissural axon guidance in the zebrafish forebrain. PLoS ONE 12:e0176036. 10.1371/journal.pone.0176036 28448525 PMC5407822

[B127] Mohanasundaram P. Shanmugam M. M. (2010). Role of syntaxin 4 in activity-dependent exocytosis and synaptic plasticity in hippocampal neurons. Sci. Signal. 3:jc7. 10.1126/scisignal.3144jc7 20959521

[B128] Moschner K. Sündermann F. Meyer H. da Graca A. P. Appel N. Paululat A.. (2014). RNA protein granules modulate tau isoform expression and induce neuronal sprouting. J. Biol. Chem. 289, 16814–16825. 10.1074/jbc.M113.541425 24755223 PMC4059124

[B129] Myers J. P. Santiago-Medina M. Gomez T. M. (2011). Regulation of axonal outgrowth and pathfinding by integrin-ECM interactions. Dev. Neurobiol. 71, 901–923. 10.1002/dneu.20931 21714101 PMC3192254

[B130] Nagao M. Lanjakornsiripan D. Itoh Y. Kishi Y. Ogata T. Gotoh Y. (2014). High mobility group nucleosome-binding family proteins promote astrocyte differentiation of neural precursor cells. Stem Cells 32, 2983–2997. 10.1002/stem.1787 25069414

[B131] Nakatani Y. Konishi H. Vassilev A. Kurooka H. Ishiguro K. Sawada J.. (2005). p600, a unique protein required for membrane morphogenesis and cell survival. Proc. Natl. Acad. Sci. U.S.A. 102, 15093–15098. 10.1073/pnas.0507458102 16214886 PMC1247991

[B132] Namadurai S. Yereddi N. R. Cusdin F. S. Huang C. L. H. Chirgadze D. Y. Jackson A. P. (2015). A new look at sodium channel β subunits. Open Biol. 5:140192. 10.1098/rsob.140192 25567098 PMC4313373

[B133] Ney A. Booms P. Epple G. Mörgelin M. Guo G. Kettelgerdes G.. (2006). Calcium-dependent self-association of the C-type lectin domain of versican. Int. J. Biochem. Cell Biol. 38, 23–29. 10.1016/j.biocel.2005.07.007 16159712

[B134] Park J. Kim D. H. Kim H. N. Wang C. J. Kwak M. K. Hur E.. (2016). Directed migration of cancer cells guided by the graded texture of the underlying matrix. Nat. Mater. 15, 792–801. 10.1038/nmat4586 26974411 PMC5517090

[B135] Parker E. M. Monopoli A. Ongini E. Lozza G. Babij C. M. (2000). Rapamycin, but not FK506 and GPI-1046, increases neurite outgrowth in PC12 cells by inhibiting cell cycle progression. Neuropharmacology 39, 1913–1919. 10.1016/S0028-3908(00)00028-9 10884572

[B136] Parsons K. Nakatani Y. Nguyen M. D. (2015). p600/UBR4 in the central nervous system. Cell Mol. Life Sci. 72, 1149–1160. 10.1007/s00018-014-1788-8 25424645 PMC11113099

[B137] Paszek M. J. DuFort C. C. Rossier O. Bainer R. Mouw J. K. Godula K.. (2014). The cancer glycocalyx mechanically primes integrin-mediated growth and survival. Nature 511, 319–325. 10.1038/nature13535 25030168 PMC4487551

[B138] Perez-Reyes E. Cribbs L. L. Daud A. Lacerda A. E. Barclay J. Williamson M. P.. (1998). Molecular characterization of a neuronal low-voltage-activated T-type calcium channel. Nature 391, 896–900. 10.1038/36110 9495342

[B139] Persengiev S. P. Kondova I. I. Kilpatrick D. L. (1999). E2F4 actively promotes the initiation and maintenance of nerve growth factor-induced cell differentiation. Mol. Cell Biol. 19, 6048–6056. 10.1128/MCB.19.9.6048 10454552 PMC84505

[B140] Peterson T. R. Sengupta S. S. Harris T. E. Carmack A. E. Kang S. A. Balderas E.. (2011). mTOR complex 1 regulates lipin 1 localization to control the SREBP pathway. Cell 146, 408–420. 10.1016/j.cell.2011.06.034 21816276 PMC3336367

[B141] Pfister J. A. D'Mello S. R. (2015). Insights into the regulation of neuronal viability by nucleophosmin/B23. Exp. Biol. Med. 240, 774–786. 10.1177/1535370215579168 25908633 PMC4640450

[B142] Piao X. Hill R. S. Bodell A. Chang B. S. Basel-Vanagaite L. Straussberg R.. (2004). G protein-coupled receptor-dependent development of human frontal cortex. Science 303, 2033–2036. 10.1126/science.1092780 15044805

[B143] Podestà A. Borghi F. Indrieri M. Bovio S. Piazzoni C. Milani P. (2015). Nanomanufacturing of titania interfaces with controlled structural and functional properties by supersonic cluster beam deposition. J. Appl. Phys. 118:234309 10.1063/1.4937549

[B144] Porazinski S. Wang H. Asaoka Y. Behrndt M. Miyamoto T. Morita H.. (2015). YAP is essential for tissue tension to ensure vertebrate 3D body shape. Nature 521, 217–221. 10.1038/nature14215 25778702 PMC4720436

[B145] Potkin S. G. Turner J. A. Fallon J. A. Lakatos A. Keator D. B. Guffanti G.. (2009). Gene discovery through imaging genetics: identification of two novel genes associated with schizophrenia. Mol. Psychiatry 14, 416–428. 10.1038/mp.2008.127 19065146 PMC3254586

[B146] Pouwels J. Nevo J. Pellinen T. Ylänne J. Ivaska J. (2012). Negative regulators of integrin activity. J. Cell Sci. 125(Pt 14), 3271–3280. 10.1242/jcs.093641 22822081

[B147] Qing Y. Yingmao G. Lujun B. Shaoling L. (2008). Role of Npm1 in proliferation, apoptosis and differentiation of neural stem cells. J. Neurol. Sci. 266, 131–137. 10.1016/j.jns.2007.09.029 17949752

[B148] Racchetti G. Lorusso A. Schulte C. Gavello D. Carabelli V. D'Alessandro R.. (2010). Rapid neurite outgrowth in neurosecretory cells and neurons is sustained by the exocytosis of a cytoplasmic organelle, the enlargeosome. J. Cell Sci. 123(Pt 2), 165–170. 10.1242/jcs.059634 20026640

[B149] Raucher D. Sheetz M. P. (1999). Membrane expansion increases endocytosis rate during mitosis. J. Cell Biol. 144, 497–506. 10.1083/jcb.144.3.497 9971744 PMC2132908

[B150] Rescher U. Gerke V. (2008). S100A10/p11: family, friends and functions. Pflugers Arch. 455, 575–582. 10.1007/s00424-007-0313-4 17638009

[B151] Robertson J. Jacquemet G. Byron A. Jones M. C. Warwood S. Selley J. N.. (2015). Defining the phospho-adhesome through the phosphoproteomic analysis of integrin signalling. Nat. Commun. 6:6265. 10.1038/ncomms7265 25677187 PMC4338609

[B152] Rujano M. A. Sanchez-Pulido L. Pennetier C. le Dez G. Basto R. (2013). The microcephaly protein Asp regulates neuroepithelium morphogenesis by controlling the spatial distribution of myosin II. Nat. Cell Biol. 15, 1294–1306. 10.1038/ncb2858 24142104

[B153] Sarhan A. R. Patel T. R. Cowell A. R. Tomlinson M. G. Hellberg C. Heath J. K.. (2016). LAR protein tyrosine phosphatase regulates focal adhesions through CDK1. J. Cell Sci. 129, 2962–2971. 10.1242/jcs.191379 27352860

[B154] Schulte C. Podestà A. Lenardi C. Tedeschi G. Milani P. (2017). Quantitative control of protein and cell interaction with nanostructured surfaces by cluster assembling. Acc. Chem. Res. 50, 231–239. 10.1021/acs.accounts.6b00433 28116907

[B155] Schulte C. Racchetti G. D'Alessandro R. Meldolesi J. (2010). A new form of neurite outgrowth sustained by the exocytosis of enlargeosomes expressed under the control of REST. Traffic 11, 1304–1314. 10.1111/j.1600-0854.2010.01095.x 20604903

[B156] Schulte C. Rodighiero S. Cappelluti M. A. Puricelli L. Maffioli E. Borghi F.. (2016a). Conversion of nanoscale topographical information of cluster-assembled zirconia surfaces into mechanotransductive events promotes neuronal differentiation. J. Nanobiotechnol. 14:18. 10.1186/s12951-016-0171-3 26955876 PMC4784317

[B157] Schulte C. Ripamonti M. Maffioli E. Cappelluti M. A. Nonnis S. Puricelli L.. (2016b). Scale invariant disordered nanotopography promotes hippocampal neuron development and maturation with involvement of mechanotransductive pathways. Front. Cell Neurosci. 10:267. 10.3389/fncel.2016.00267 27917111 PMC5114288

[B158] Schulte C. Ferraris G. M. S. Oldani A. Galluzzi M. Podestà A. Puricelli L. (2016c). Lamellipodial tension, not integrin/ligand binding, is the crucial factor to realise integrin activation and cell migration. Eur. J. Cell Biol. 95, 1–14. 10.1016/j.ejcb.2015.10.002 26616200

[B159] Selak S. Paternain A. V. Aller M. I. Aller I. M. Picó E. Rivera R.. (2009). A role for SNAP25 in internalization of kainate receptors and synaptic plasticity. Neuron. 63, 357–371. 10.1016/j.neuron.2009.07.017 19679075

[B160] Shen B. Delaney M. K. Du X. (2012). Inside-out, outside-in, and inside-outside-in: G protein signaling in integrin-mediated cell adhesion, spreading, and retraction. Curr. Opin. Cell Biol. 24, 600–606. 10.1016/j.ceb.2012.08.011 22980731 PMC3479359

[B161] Sinha B. Köster D. Ruez R. Gonnord P. Bastiani M. Abankwa D.. (2011). Cells respond to mechanical stress by rapid disassembly of caveolae. Cell 144, 402–413. 10.1016/j.cell.2010.12.031 21295700 PMC3042189

[B162] Spangler S. A. Hoogenraad C. C. (2007). Liprin-alpha proteins: scaffold molecules for synapse maturation. Biochem. Soc. Trans. 35(Pt 5), 1278–1282. 10.1042/BST0351278 17956329

[B163] Spiliotis E. T. Nelson W. J. (2006). Here come the septins: novel polymers that coordinate intracellular functions and organization. J. Cell Sci. 119, 4–10. 10.1242/jcs.02746 16371649 PMC3368708

[B164] Stevens B. Allen N. J. Vazquez L. E. Howell G. R. Christopherson K. S. Nouri N.. (2007). The classical complement cascade mediates CNS synapse elimination. Cell 131, 1164–1178. 10.1016/j.cell.2007.10.036 18083105

[B165] Sukharev S. Sachs F. (2012). Molecular force transduction by ion channels: diversity and unifying principles. J. Cell Sci. 125(Pt 13), 3075–3083. 10.1242/jcs.092353 22797911 PMC3434843

[B166] Swift J. Ivanovska I. L. Buxboim A. Harada T. Dingal P. C. Pinter J.. (2013). Nuclear lamin-A scales with tissue stiffness and enhances matrix-directed differentiation. Science 341:1240104. 10.1126/science.1240104 23990565 PMC3976548

[B167] Takamori Y. Tamura Y. Kataoka Y. Cui Y. Seo S. Kanazawa T.. (2007). Differential expression of nuclear lamin, the major component of nuclear lamina, during neurogenesis in two germinal regions of adult rat brain. Eur. J. Neurosci. 25, 1653–1662. 10.1111/j.1460-9568.2007.05450.x 17432957

[B168] Tamplenizza M. Lenardi C. Maffioli E. Nonnis S. Negri A. Forti S.. (2013). Nitric oxide synthase mediates PC12 differentiation induced by the surface topography of nanostructured TiO2. J. Nanobiotechnol. 11:35. 10.1186/1477-3155-11-35 24119372 PMC3815074

[B169] Tan C. L. Kwok J. C. Heller J. P. Zhao R. Eva R. Fawcett J. W. (2015). Full length talin stimulates integrin activation and axon regeneration. Mol. Cell Neurosci. 68:1–8. 10.1016/j.mcn.2015.03.011 25771432 PMC4604251

[B170] Tang F.-L. Liu W. Hu J.-X. Erion J. R. Ye J. Mei L. (2015). VPS35 deficiency or mutation causes dopaminergic neuronal loss by impairing mitochondrial fusion and function. Cell Rep. 12, 1631–1643. 10.1016/j.celrep.2015.08.001 26321632 PMC4565770

[B171] Tennstaedt A. Pöpsel S. Truebestein L. Hauske P. Brockmann A. Schmidt N.. (2012). Human high temperature requirement serine protease A1 (HTRA1) degrades tau protein aggregates. J. Biol. Chem. 287, 20931–20941. 10.1074/jbc.M111.316232 22535953 PMC3375517

[B172] Thomanetz V. Angliker N. Cloëtta D. Lustenberger R. M. Schweighauser M. Oliveri F.. (2013). Ablation of the mTORC2 component rictor in brain or Purkinje cells affects size and neuron morphology. J. Cell Biol. 201, 293–308. 10.1083/jcb.201205030 23569215 PMC3628512

[B173] Thomson S. E. Charalambous C. Smith C. A. Tsimbouri P. M. Déjardin T. Kingham P. J.. (2017). Microtopographical cues promote peripheral nerve regeneration via transient mTORC2 activation. Acta Biomater. 60, 220–231. 10.1016/j.actbio.2017.07.031 28754648 PMC5593812

[B174] Toffolo E. Rusconi F. Paganini L. Tortorici M. Pilotto S. Heise C.. (2014). Phosphorylation of neuronal Lysine-Specific Demethylase 1LSD1/KDM1A impairs transcriptional repression by regulating interaction with CoREST and histone deacetylases HDAC1/2. J. Neurochem. 128, 603–616. 10.1111/jnc.12457 24111946

[B175] Toth A. B. Shum A. K. Prakriya M. (2016). Regulation of neurogenesis by calcium signaling. Cell Calcium 59, 124–34. 10.1016/j.ceca.2016.02.011 27020657 PMC5228525

[B176] Um J. W. Ko J. (2013). LAR-RPTPs: synaptic adhesion molecules that shape synapse development. Trends Cell Biol. 23, 465–475. 10.1016/j.tcb.2013.07.004 23916315

[B177] Vasudevan A. Ho M. S. P. Weiergräber M. Nischt R. Schneider T. Lie A.. (2010). Basement membrane protein nidogen-1 shapes hippocampal synaptic plasticity and excitability. Hippocampus 20, 608–620. 10.1002/hipo.20660 19530222

[B178] Vizcaíno J. A. Csordas A. del-Toro N. Dianes J. A. Griss J. Lavidas I. (2016). update of the PRIDE database and its related tools. Nucleic Acids Res. 44, D447–D456. 10.1093/nar/gkw880 26527722 PMC4702828

[B179] Voegel J. J. Heine M. J. Zechel C. Chambon P. Gronemeyer H. (1996). TIF2, a 160 kDa transcriptional mediator for the ligand-dependent activation function AF-2 of nuclear receptors. EMBO J. 15, 3667–3675. 8670870 PMC452006

[B180] Vogel V. Sheetz M. (2006). Local force and geometry sensing regulate cell functions. Nat. Rev. Mol. Cell Biol. 7, 265–275. 10.1038/nrm1890 16607289

[B181] Wang C. L. Tang F. L. Peng Y. Shen C. Y. Mei L. Xiong W. C. (2012). VPS35 regulates developing mouse hippocampal neuronal morphogenesis by promoting retrograde trafficking of BACE1. Biol. Open 1, 1248–1257. 10.1242/bio.20122451 23259059 PMC3522886

[B182] Wang N. Tytell J. D. Ingber D. E. (2009). Mechanotransduction at a distance: mechanically coupling the extracellular matrix with the nucleus. Nat. Rev. Mol. Cell Biol. 10, 75–82. 10.1038/nrm2594 19197334

[B183] Wang T. Hauswirth A. G. Tong A. Dickman D. Davis G. W. (2014). Endostatin is a Trans-Synaptic signal for Homeostatic Synaptic Plasticity. Neuron 83, 616–629. 10.1016/j.neuron.2014.07.003 25066085 PMC4133507

[B184] Wegner K. Piseri P. Tafreshi H. V. Milani P. (2006). Cluster beam deposition: a tool for nanoscale science and technology. J. Phys. Appl. Phys. 39:R439 10.1088/0022-3727/39/22/R02

[B185] Wiszniak S. Kabbara S. Lumb R. Scherer M. Secker G. Harvey N.. (2013). The ubiquitin ligase Nedd4 regulates craniofacial development by promoting cranial neural crest cell survival and stem-cell like properties. Dev. Biol. 383, 186–200. 10.1016/j.ydbio.2013.09.024 24080509

[B186] Wu X. Jin W. Liu X. Fu H. Gong P. Xu J.. (2012). Cyclic AMP response element modulator-1 (CREM-1) involves in Neuronal Apoptosis after Traumatic brain injury. J. Mol. Neurosci. 47, 357–367. 10.1007/s12031-012-9761-1 22569987

[B187] Wu Y. Sheng W. Chen L. Dong H. Lee V. Lu F.. (2004). Versican V1 isoform induces neuronal differentiation and promotes neurite outgrowth. Mol. Biol. Cell 15, 2093–2104. 10.1091/mbc.E03-09-0667 14978219 PMC404007

[B188] Wyszynski M. Kim E. Dunah A. W. Passafaro M. Valtschanoff J. G. Serra-Pagès C.. (2002). Interaction between GRIP and liprin-alpha/SYD2 is required for AMPA receptor targeting. Neuron 34, 39–52. 10.1016/S0896-6273(02)00640-2 11931740

[B189] Xie J. Proud C. G. (2013). Crosstalk between mTOR complexes. Nat. Cell Biol. 15, 1263–1265. 10.1038/ncb2877 24189516

[B190] Xu J. Xiao N. Xia J. (2010). Thrombospondin 1 accelerates synaptogenesis in hippocampal neurons through neuroligin 1. Nat. Neurosci. 13, 22–24. 10.1038/nn.2459 19915562

[B191] Yamaguchi Y. Katoh H. Yasui H. Mori K. Negishi M. (2001). RhoA inhibits the nerve growth factor-induced Rac1 activation through Rho-associated kinase-dependent pathway. J. Biol. Chem. 276, 18977–18983. 10.1074/jbc.M100254200 11279039

[B192] Yudin D. Fainzilber M. (2009). Ran on tracks – cytoplasmic roles for a nuclear regulator. J. Cell Sci. 122, 587–593. 10.1242/jcs.015289 19225125

[B193] Zakaria S. Mao Y. Kuta A. de Sousa C. F. Gaufo G. O. McNeill H.. (2014). Regulation of neuronal migration by Dchs1-Fat4 planar cell polarity. Curr. Biol. 24, 1620–1627. 10.1016/j.cub.2014.05.067 24998526 PMC4193925

[B194] Zamboni V. Armentano M. Sarò G. Ciraolo E. Ghigo A. Germena G.. (2016). Disruption of ArhGAP15 results in hyperactive Rac1, affects the architecture and function of hippocampal inhibitory neurons and causes cognitive deficits. Sci. Rep. 6:34877. 10.1038/srep34877 27713499 PMC5054378

[B195] Zanotti L. Angioni R. Calì B. Soldani C. Ploia C. Moalli F.. (2016). Mouse mesenchymal stem cells inhibit high endothelial cell activation and lymphocyte homing to lymph nodes by releasing TIMP-1. Leukemia 30, 1143–1154. 10.1038/leu.2016.33 26898191 PMC4858586

[B196] Zhang J. Abdel-Rahman A. A. (2006). Nischarin as a functional imidazoline (I1) receptor. FEBS Lett. 580, 3070–3074. 10.1016/j.febslet.2006.04.058 16678176

[B197] Zhang K. Chen J. (2012). The regulation of integrin function by divalent cations. Cell Adhes. Migr. 6, 20–29. 10.4161/cam.18702 22647937 PMC3364134

[B198] Zhou L. Lim Q.-E. Wan G. Too H.-P. (2010). Normalization with genes encoding ribosomal proteins but not GAPDH provides an accurate quantification of gene expressions in neuronal differentiation of PC12 cells. BMC Genomics. 11:75 10.1186/1471-2164-11-75 20113474 PMC2831847

[B199] Zick Y. Eisenstein M. Goren R. A. Hadari Y. R. Levy Y. Ronen D. (2004). Role of galectin-8 as a modulator of cell adhesion and cell growth. Glycoconj. J. 19, 517–526. 10.1023/B:GLYC.0000014081.55445.af 14758075

