# Supplementary figures and images for: Proteomic Dissection of Nanotopography-Sensitive Mechanotransductive Signaling Hubs that Foster Neuronal Differentiation in PC12 Cells
Source: Front Cell Neurosci. 2018 Jan 4;11:417. doi: 10.3389/fncel.2017.00417 (PMC5758595; doi:10.3389/fncel.2017.00417)

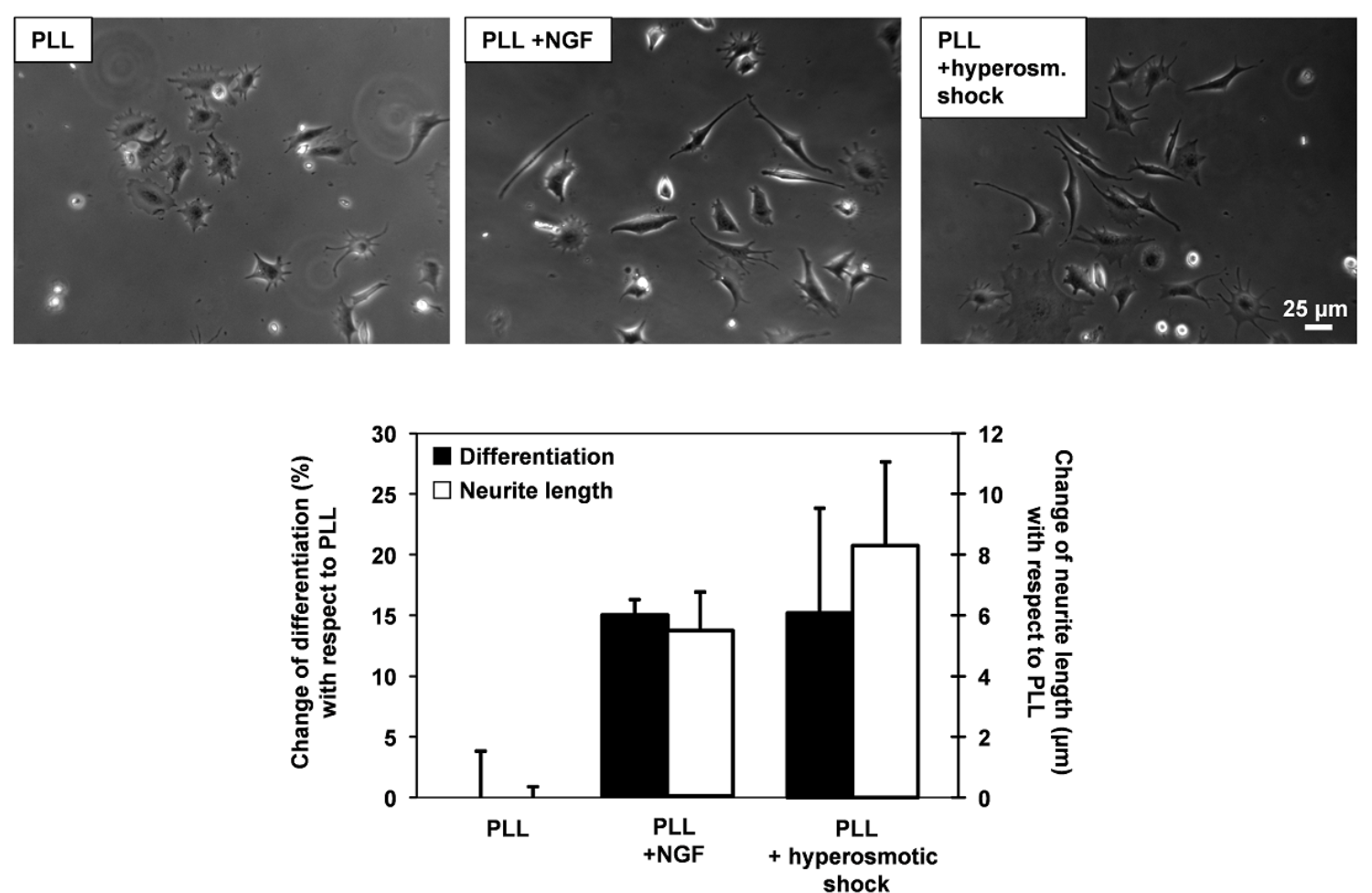

Supplement: Figure S1 — Induction of neurite outgrowth in PC12 cells by hyperosmotic shock on PLL. The phase contrast images in the panel illustrate the response of PC12 cells on PLL after applying a hyperosmotic shock (15 min, 150 mM sucrose), with the conditions PLL ± NGF as references. The graph represents the global statistic of two independent experiments (in total 343–398 cells and 226–251 neurites quantified). The bars show the average (mean) change compared to the PLL –NGF condition (white bars: differentiation rate; black bars: neurite length) and are flanked by the standard deviation (s.d.). [file Image1.TIF]

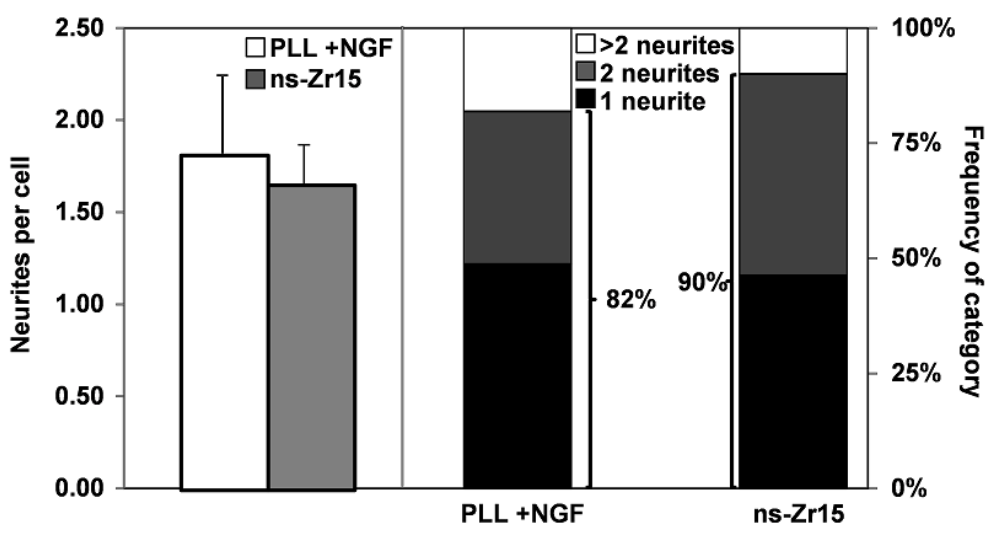

Supplement: Figure S2 — Comparison of the neurite morphology of PC12 cells in the between NGF and ns-Zr15 condition. The graph shows on the left the average number neurites per differentiated PC12 cell grown on PLL and stimulated with NGF (white bar) or interacting with ns-Zr15 (gray bar). The bars are flanked by the standard deviation (s.d.). On the right, the bars demonstrate the frequency of the different categories indicating the number of neurites per differentiated cells in the two conditions. Representative examples of the cell morphology in these conditions can be found in the phase contrast images of Figures 1, 5, 6. In total 160 cells from 8 independent experiments were quantified. [file Image2.TIF]

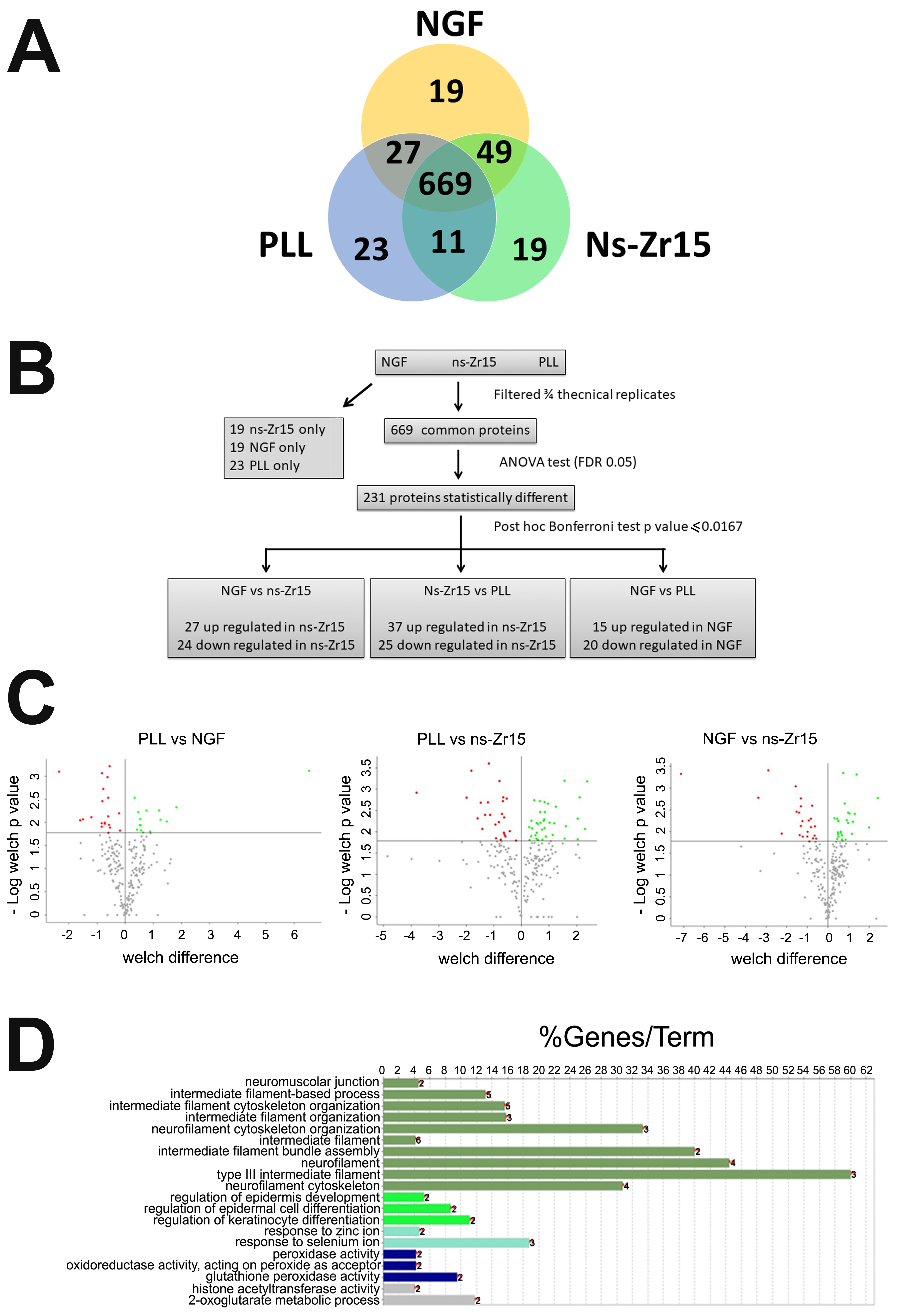

Supplement: Figure S3 — Comparison of PLL, NGF and ns-Zr15. (A) Venn diagram; (B) work flow; (C) Vulcano plots; (D) ClueGo analysis of proteins upregulated or expressed only in cells grown on ns-Zr15vsNGF. Functional grouping was based on p ≤ 0.05 and at least two counts. [file Image3.TIF]

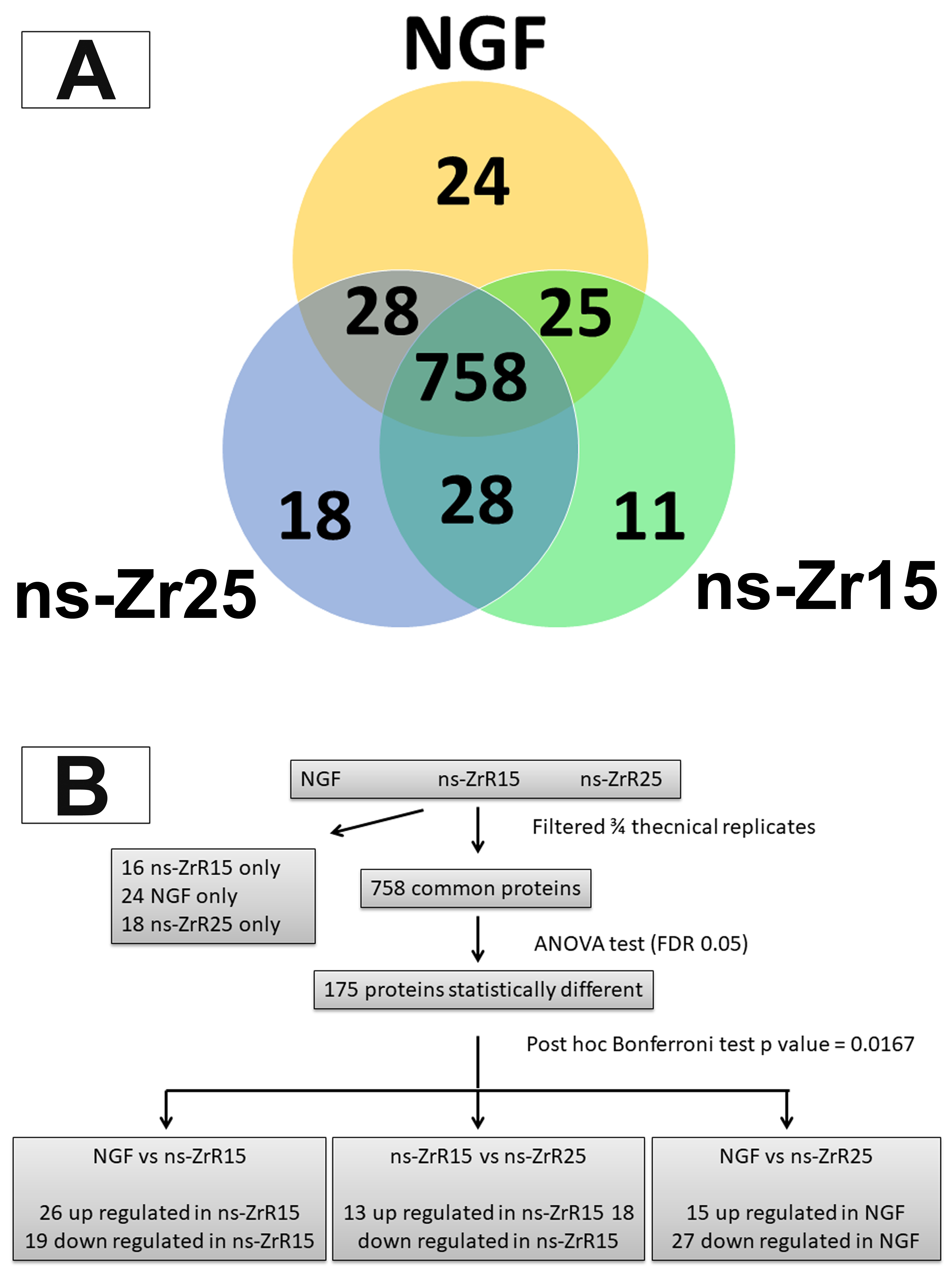

Supplement: Figure S4 — Comparison of NGF, ns-Zr15, ns-Zr25. (A) Venn diagram; (B) work flow [file Image4.TIF]
